# Supplementary figures and images for: Centrosome amplification primes ovarian cancer cells for apoptosis and potentiates the response to chemotherapy
Source: PLoS Biol. 2024 Sep 5;22(9):e3002759. doi: 10.1371/journal.pbio.3002759 (PMC11441705; doi:10.1371/journal.pbio.3002759)

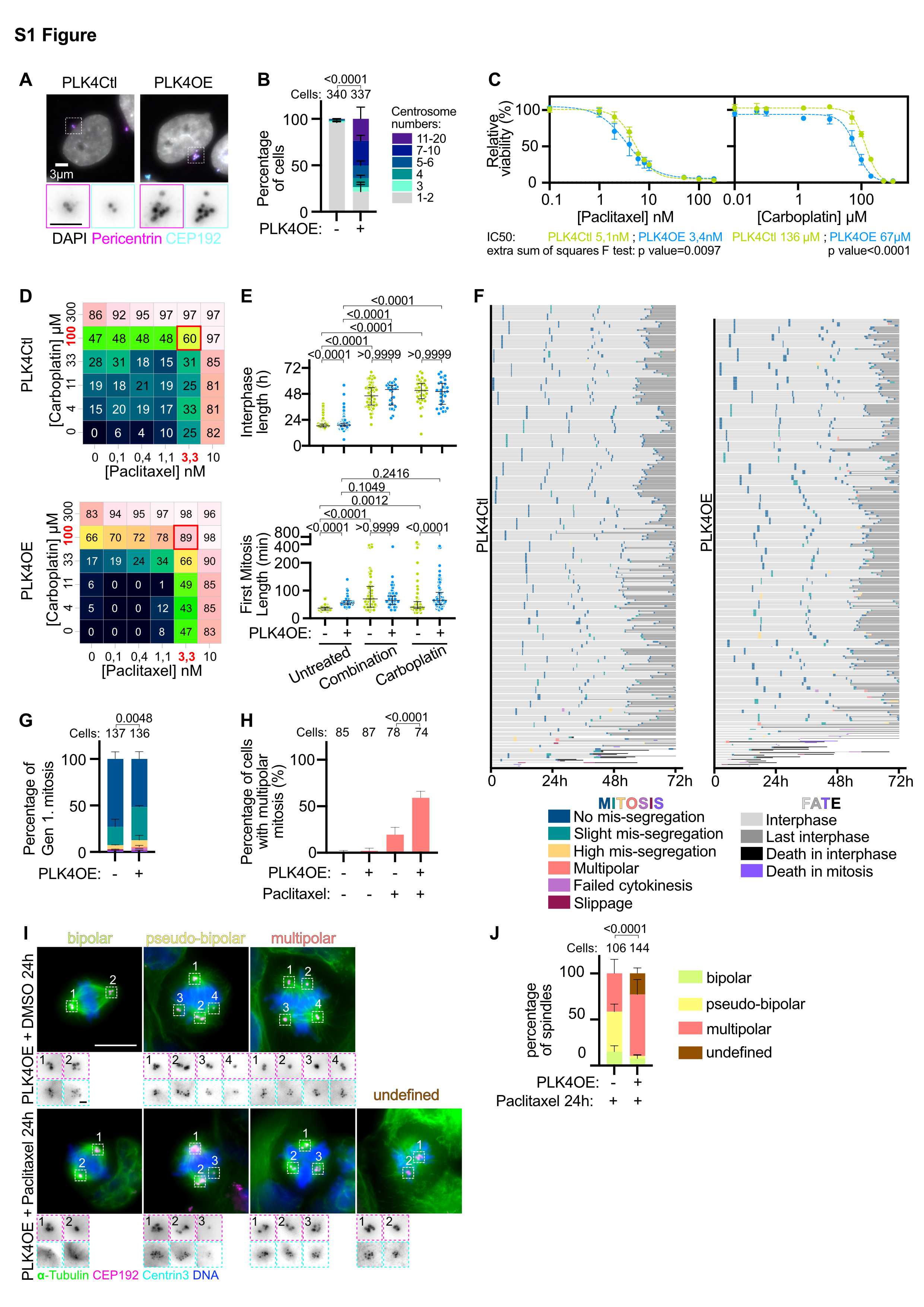

Supplement: S1 Fig — (A) Representative images of OVCAR8 cells stained with DAPI (gray) and antibodies against CEP192 (Cyan) and Pericentrin (Magenta). (B) Bar graphs showing the averages and SEM of the percentage of cells with the indicated number of centrosomes (CEP192 dots colocalizing with Pericentrin). Three independent experiments, statistical test: Fisher’s exact test comparing the number of cells with more than 2 centrosomes. (C) Dose-response of PLK4Ctl and PLK4OE cells to Paclitaxel (left) and Carboplatin (right), normalized to their respective control conditions, obtained from MTT viability assays. Mean and SEM of 2 independent experiments each obtained from averaging 3 technical replicates. (D) Combination matrixes for Carboplatin and Paclitaxel combined treatment, representing percentage of viability inhibition compared to control cells. Chosen working concentrations are highlighted in red. (E) Scatter dot plots of Interphase length (top) and First mitosis length (bottom), with Median and interquartile range. Data from 2 independent experiments are pooled for Combined treatment and Carboplatin treatment, data from the 4 corresponding control experiments are pooled for Untreated. For interphase length a minimum of 26 cells was analyzed, and for mitosis length a minimum of 133 cells was analyzed. Statistical tests: Kruskal–Wallis with Dunn’s multiple comparisons tests. (F) Single-cell profiles of PLK4Ctl (left) and PLK4OE (right) Untreated cells. Color coding of mitosis and fates refers to categories defined in Fig 1A. (G) Averages and SEM of the percentages of mitotic phenotypes (legends in Fig 1A and 1F). Two independent experiments, statistical test: Fisher’s exact test on the number of Slight Mis-segregation events. (H) Percentage of multipolar divisions observed in presence or absence of 5 nM Paclitaxel. Two independent experiments, statistical test: Fisher’s exact test on the number of multipolar divisions. (I) Representative images of OVCAR8 cells in prometaphase/metap [file pbio.3002759.s001.tiff]

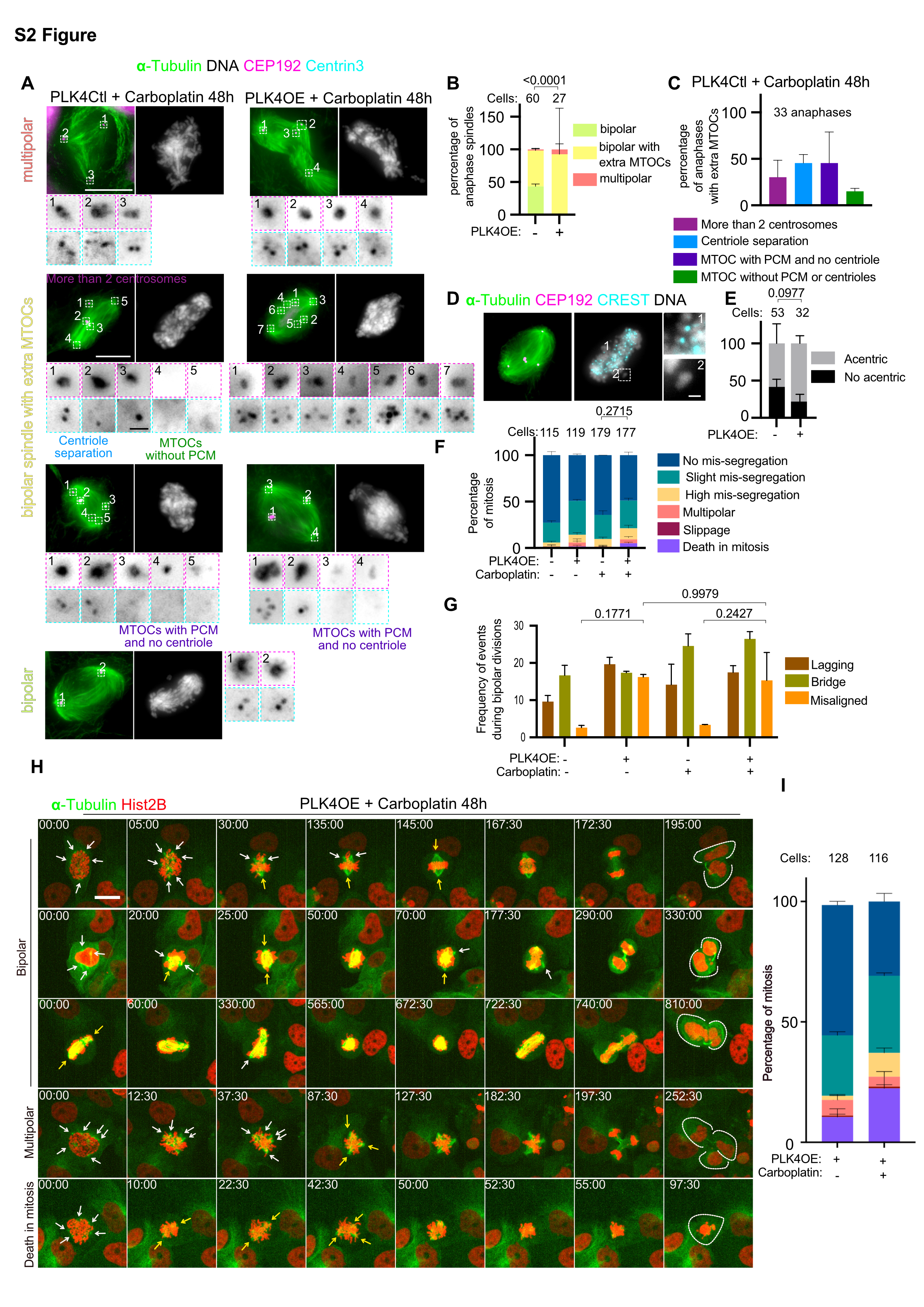

Supplement: S2 Fig — (A) Representative images of OVCAR8 cells in anaphase stained with DAPI (gray), and antibodies against α-Tubulin (green), CEP192 (magenta) and Centrin3 (cyan). Numbered insets to show centrosomes and spindle poles are presented below the spindle image. Scale bars are 10 μm for spindle images and 1 μm for centrosome insets. (B) Averages and SEM of the percentages of spindle types shown in panel C. Two independent experiments, statistical test: Fisher’s exact test on the number of bipolar spindles. (C) Averages and SEM of the percentages of Carboplatin treated PLK4Ctl cells presenting additional MTOCs with the indicated characteristics. (D) Representative image of a Carboplatin treated PLK4OE OVCAR8 cell in anaphase stained with DAPI (gray), and antibodies against α-Tubulin (green), CEP192 (magenta) and CREST (cyan). Numbered insets to show chromosomes with (1) or without (2) centromeres. Scale bars are 10 μm for spindle image and 1 μm for chromosome insets. (E) Averages and SEM of the percentages of mitotic figures depending on the presence of acentric chromosomes. Two independent experiments, statistical test: Fisher’s exact test on the total number of bipolar and pseudo-bipolar spindles. (F) Averages and SEM of the percentages of mitotic behaviours. Two independent experiments, statistical test: Fisher’s exact test on the number of High mis-segregation events. (G) Averages and SEM of the percentages of the indicated mis-segregation events within bipolar divisions. Two independent experiments, statistical test: ANOVA with Sidak’s multiple comparison test. (H) Time lapse stills of iOVCAR8 cells expressing GFP-Tubulin (green) and H2B-RFP (red) to visualize the mitotic spindle and chromosomes treated with DOX and carboplatin. Time is shown in minutes. White arrows point to centrosomes that do not show a clustering behavior, while yellow arrows point to clustered centrosomes. In the top row, a cell enters mitosis with at least 5 centrosomes, it goes through a multipolar [file pbio.3002759.s002.tiff]

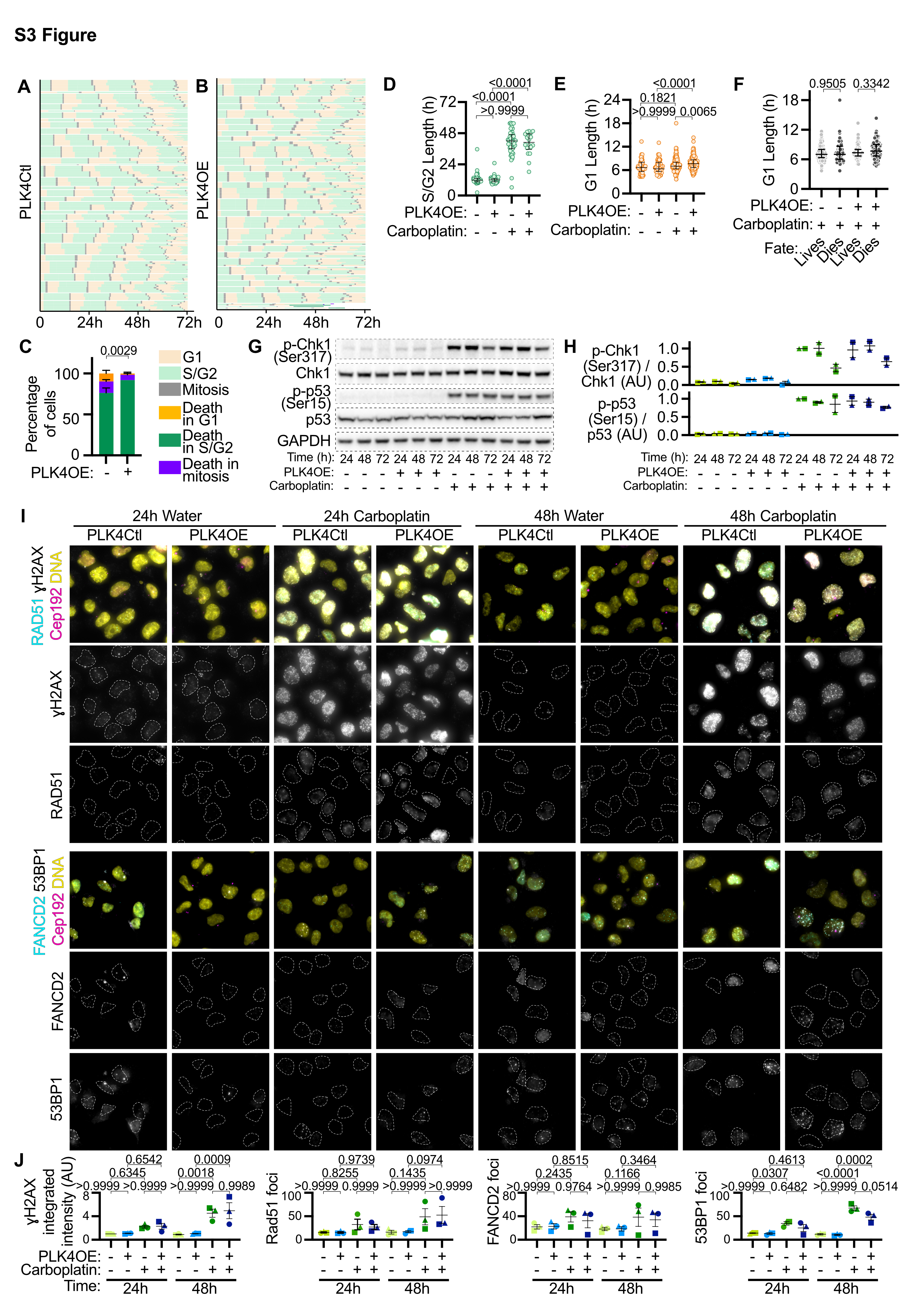

Supplement: S3 Fig — (A, B) Single-cell profiles of FUCCI PLK4Ctl (A) and PLK4OE (B) untreated cells. See panel C for color-coded legends of cell cycle phase and cell fate. (C) Bar graphs showing the averages and SEM of the percentages of cell death events occurring in the indicated cell-cycle phases. Two independent experiments, statistical test: Fisher’s exact test on number of death events occurring in S/G2. (D, E) Scatter dot plot graph of S/G2 (D) and G1 (E) phase lengths in the second generation, with median and interquartile range. Two independent experiments with a minimum of 18 times analyzed per category. Statistical tests: Kruskal–Wallis with Dunn’s multiple comparisons tests. (F) Scatter dot plot graph of G1 phase length in the second generation, with median and interquartile range, depending on the fate of the cells in the second generation. Two independent experiments with a minimum of 70 times analyzed per category. Statistical tests: Kruskal–Wallis with Dunn’s multiple comparisons tests. (G) Representative images of western blot analysis of phosphorylated Chk1 and p53. (H) Graph showing the average and SEM of phosphorylated protein relative to total protein levels, normalized to the levels detected in PLK4OE cells treated with Carboplatin for 24 h, from 2 independent experiments. (I) Representative images of cells stained with DAPI an antibodies against FANCD2 (cyan), 53BP1 (gray), and CEP192 (magenta). Grayscale images of RAD51 and γH2AX are shown. (J) Dot-plot representing integrated nuclear γH2AX fluorescence intensity per cell or numbers of Rad51, FANCD2, or 53BP1 foci per cells. Average and SEM of the averages obtained from 3 independent experiments, each quantifying a minimum of 94 cells per condition. Values are normalized to the average of untreated PLK4Ctl cells at 24 h. Statistical test: ANOVA with Sidak’s multiple comparison tests. Data for S3 Fig can be found in S7 Data. (TIFF) [file pbio.3002759.s003.tiff]

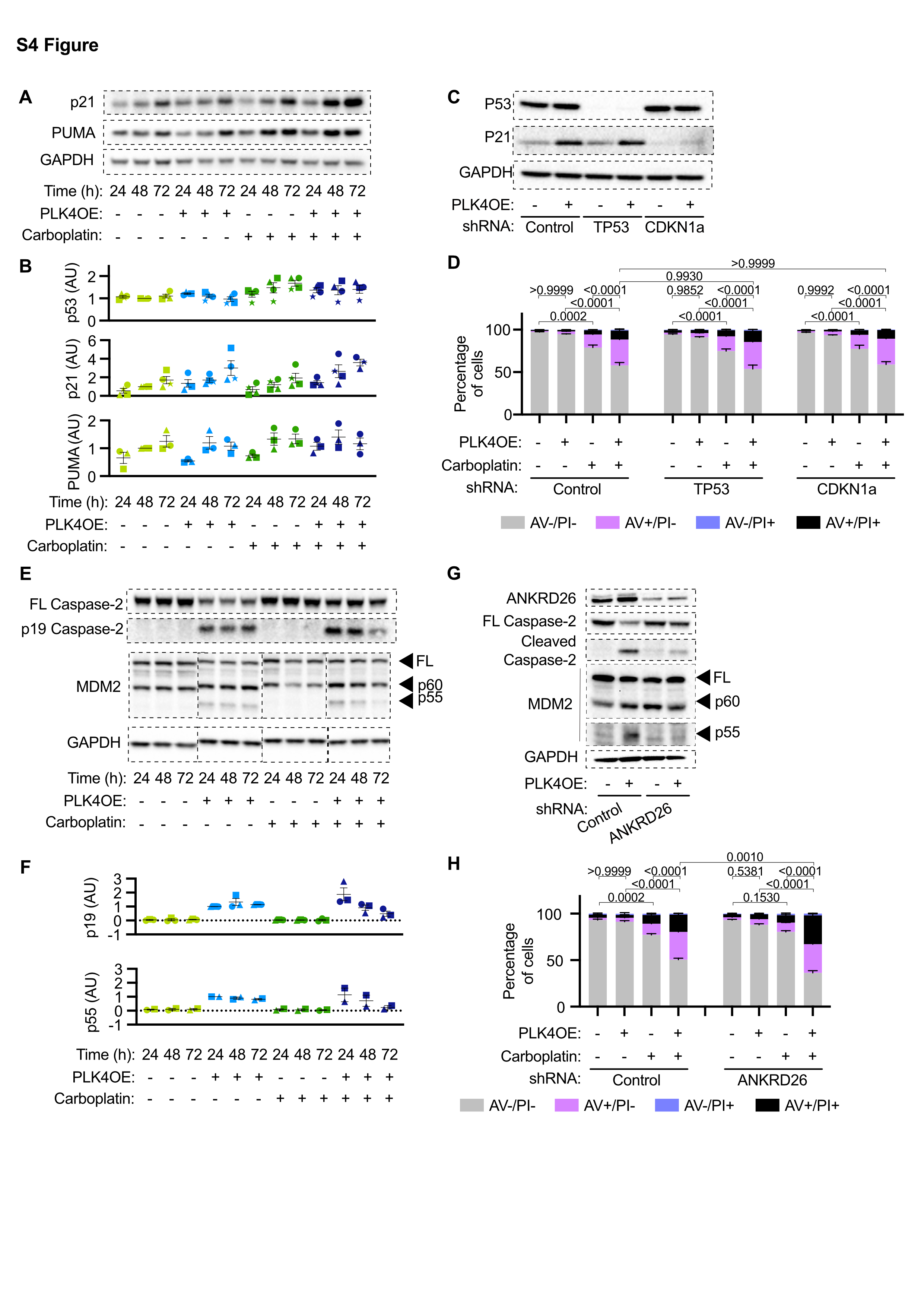

Supplement: S4 Fig — (A) Representative images of western blot analysis of p21 and PUMA. (B) Graph showing the average and SEM of protein levels from 4 (p53 and p21) or 3 (PUMA) independent experiments, normalized to levels measured in untreated PLK4Ctl cells at 48 h. (C and G) Representative images of western blot analysis of indicated shRNA cell lines. (D and H) Bar graphs showing the average and SEM of the percentage of cells in specified Annexin V-APC/PI gates analyzed by flow cytometry. (D) Six replicates obtained from 4 independent experiments, with a minimum of 10,000 cells analyzed per condition and replicate. (H) Four replicates obtained from 2 independent experiments, with a minimum of 10,000 cells analyzed per condition and replicate. Statistical test: comparison of the percentage of Annexin V positive cells, using ANOVA with Sidak’s multiple comparison test. Representative cytometry profiles can be found in the S1 Appendix. (E) Representative images of western blot analysis of Caspase2 and MDM2 cleavage. (F) Average and SEM of protein levels from 3 (p19 Caspase2) or 2 (p55 MDM2) independent experiments, normalized to levels measured in untreated PLK4OE cells at 24 h. Data for S4 Fig can be found in S8 Data. (TIFF) [file pbio.3002759.s004.tiff]

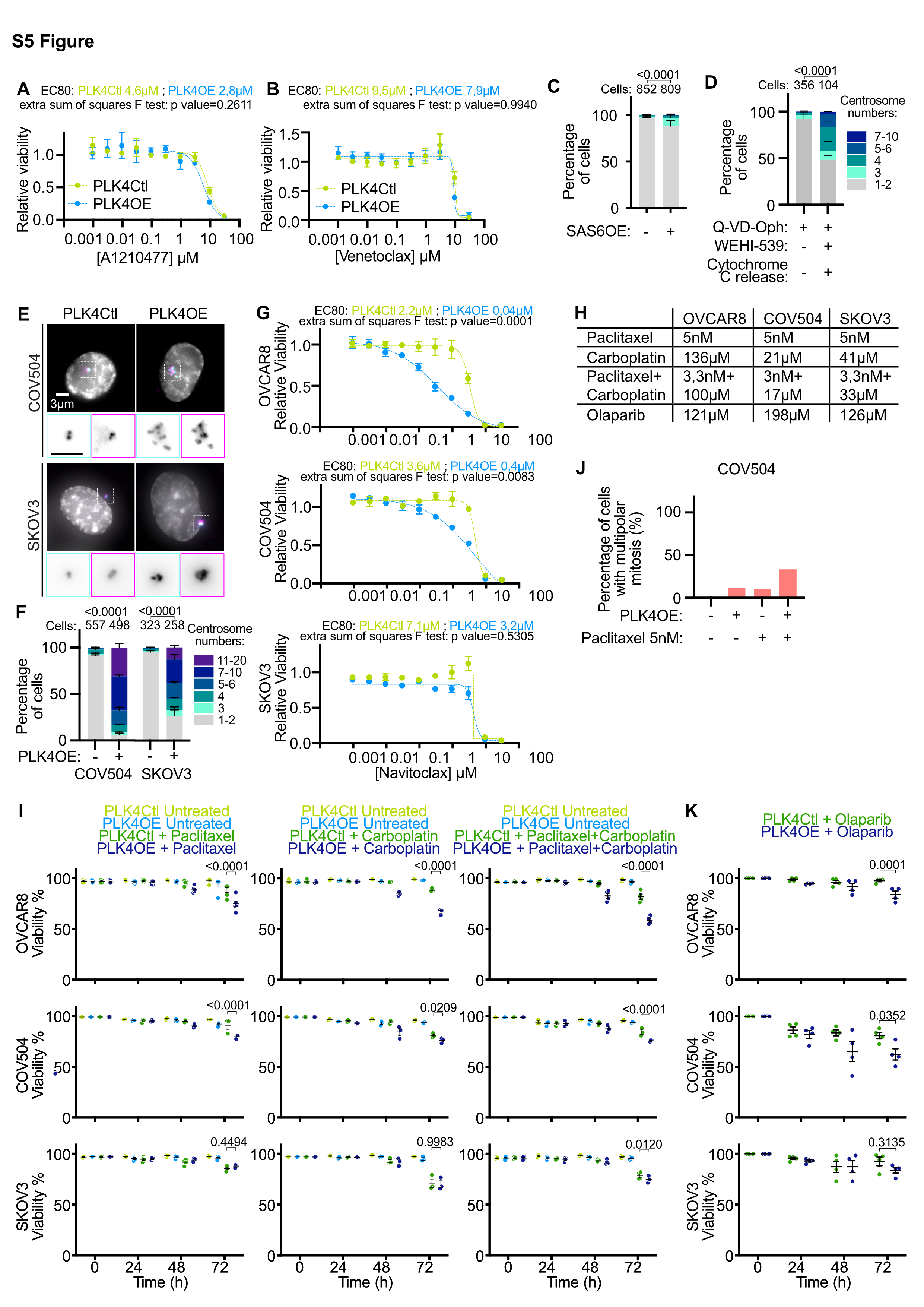

Supplement: S5 Fig — (A, B) Dose-response of PLK4Ctl and PLK4OE cells to A1210477 (A) and Venetoclax (B), normalized to their respective untreated conditions, obtained from MTT viability assays. Mean and SEM of 2 independent experiments each obtained from averaging 3 technical replicates. (C) Bar graphs showing the average and SEM of the percentage of cells with the indicated number of centrosomes (CEP192 dots colocalizing with Pericentrin). Two independent experiments, statistical test: Fisher’s exact test comparing the number of cells with more than 2 centrosomes. Numbers on the top of each graph represent the number of cells analyzed per condition. (D) Bar graphs showing the average and SEM of the percentage of cells with the indicated number of centrosomes (CEP192 dots colocalizing with Pericentrin). Two independent experiments, statistical test: Fisher’s exact test comparing the number of cells with more than 2 centrosomes. Numbers on the top of each graph represent the number of cells analyzed per condition. (E) Representative images of COV504 and SKOV3 cells stained with DAPI (gray) and antibodies against CEP192 (cyan) and Pericentrin (magenta). (F) Bar graphs showing the average and SEM of the percentages of cells with the indicated number of centrosomes (CEP192 dots colocalizing with Pericentrin). Three independent experiments, statistical tests: Fisher’s exact tests comparing the number of cells with more than 2 centrosomes. Numbers on the top of each graph represent the number of cells analyzed per condition. (G) Dose-response of PLK4Ctl and PLK4OE OVCAR8 (top), COV504 (middle), and SKOV3 (bottom) to Navitoclax, normalized to their respective untreated conditions, obtained from MTT viability assays. Mean and SEM of 2 to 3 independent experiments each obtained from averaging 3 technical replicates. (H) Table summarizing the IC50s and drug concentrations used in combinations, determined via MTT dose-response viability assays, for PLK4Ctl OVCAR8, COV504, and SKOV3. (I) Viability [file pbio.3002759.s005.tiff]

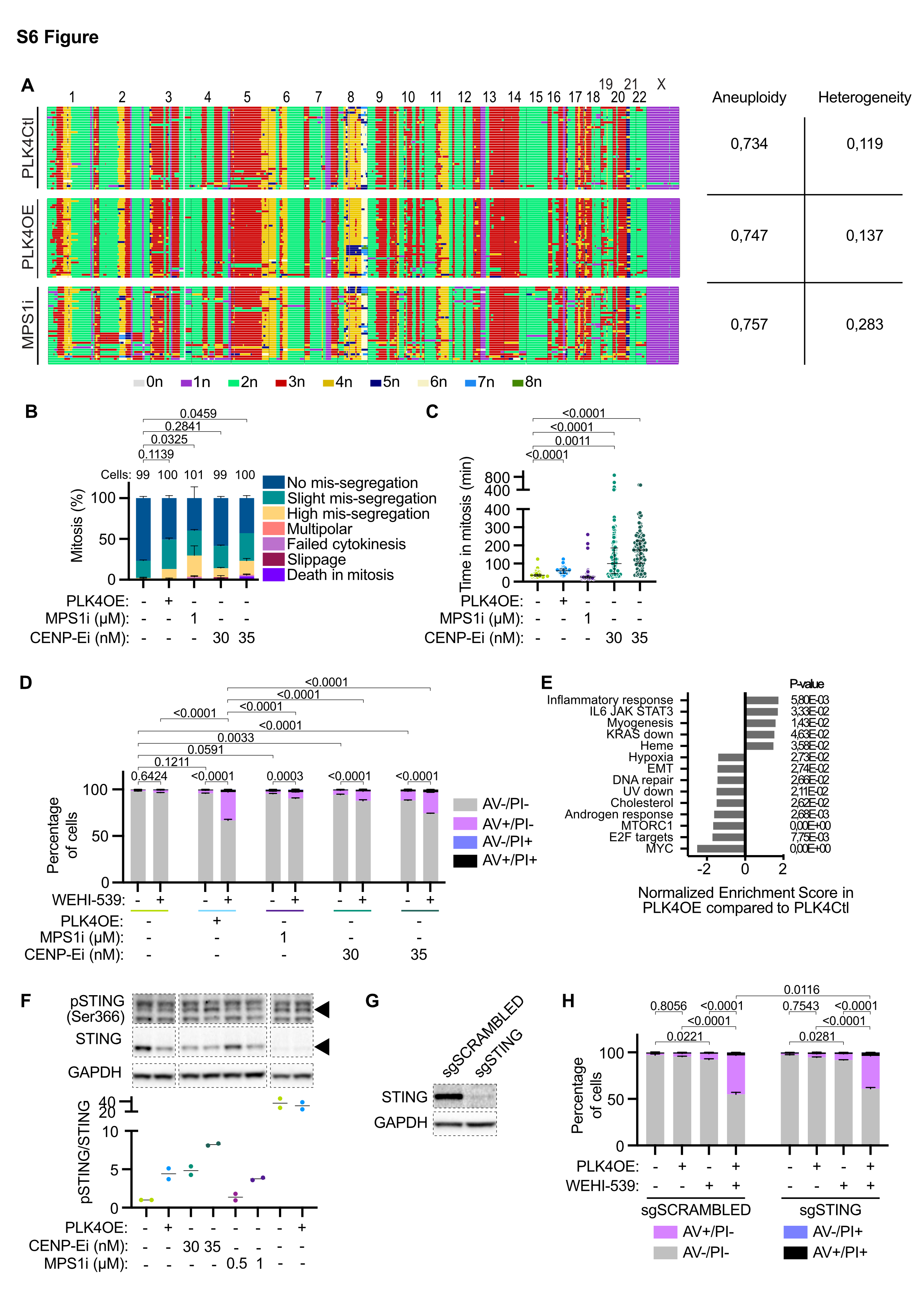

Supplement: S6 Fig — (A) Genome-wide copy-number plots for G1 OVCAR8 cells. Each row represents a cell. Indicated aneuploidy and heterogeneity scores are calculated as described in Material and methods. (B) Bar graphs showing the average and SEM of the percentage of mitotic phenotypes as defined in Fig 1A. Two independent experiments, statistical test: comparison of the percentage of cells with no mis-segregation using ANOVA with Dunnett’s multiple comparison test. Numbers on the top of each graph represent the number of cells analyzed per condition. (C) Scatter dot plot graph of mitosis length with median and interquartile range. At least 96 mitosis analyzed from 2 independent experiments, statistical test: Kruskall–Wallis with Dunn’s multiple comparison test. (D) Bar graphs showing the average and SEM of the percentage of cells in specified Annexin V-APC/PI gates analyzed by flow cytometry. Three replicates obtained from 2 independent experiments, with a minimum of 20,000 cells analyzed per condition and replicate. Statistical test: comparison of the percentage of Annexin V positive cells, using ANOVA with Sidak’s multiple comparison test. Representative cytometry profiles can be found in the S1_Appendix. (E) GSEA Hallmarks with |normalized enrichment score >1,5 and p-value <0,05, from differential RNA expression analysis of PLK4OE cells compared to PLK4Ctl. (F) Western blot analysis of STING phosphorylation after 72 h of indicated drug treatments with quantification of pSTING relative to total STING. Average and SEM of 2 independent experiments, normalized to untreated PLK4Ctl. HT-DNA transfection was performed 24 h before cell collection. (G) Western blot analysis of indicated bulk LentiCRISPR cell lines. (H) Bar graphs showing the average and SEM of the percentage of cells in specified Annexin V-APC/PI gates analyzed by flow cytometry. Four replicates obtained from 2 independent experiments with a minimum of 15,000 cells analyzed per condition and replicate. Statistical test: compa [file pbio.3002759.s006.tiff]

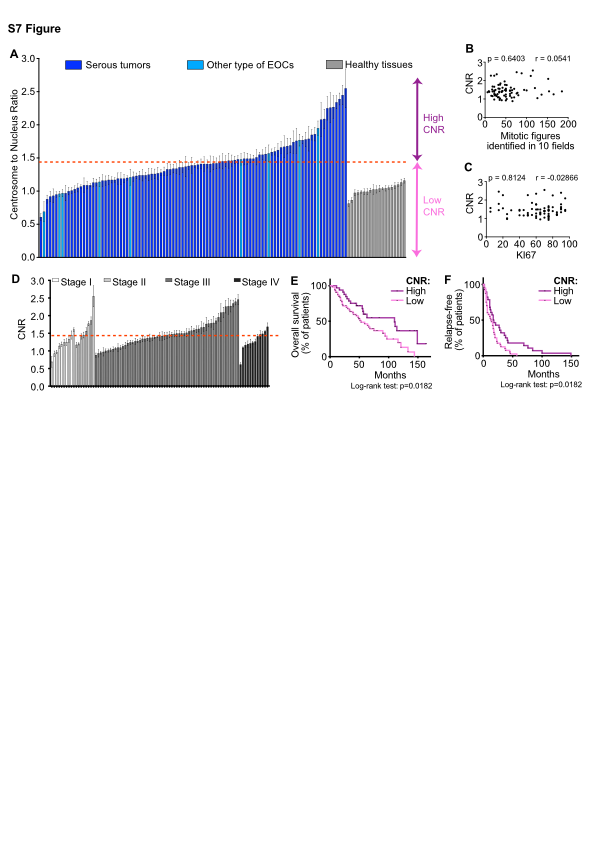

Supplement: S7 Fig — (A) Average and SEM of the CNR established in 10 fields per tumor or healthy tissue sample. Red dotted line indicates CNR = 1,45, the cut-off between High-CNR and Low-CNR patients. (B, C) Distribution of the Mitotic Index (B) and of the percentage of Ki67 positive cells (C) as a function of CNR. Statistical test: Spearman correlation. (D) Average and SEM of CNR per patient classified depending on FIGO stage. (E, F) Kaplan–Meier curves for overall survival (B) and relapse-free time after the first line of chemotherapy (C) according to CNR status. Data for S7 Fig can be found in S11 Data. (TIFF) [file pbio.3002759.s007.tiff]

S1 Appendix

3C

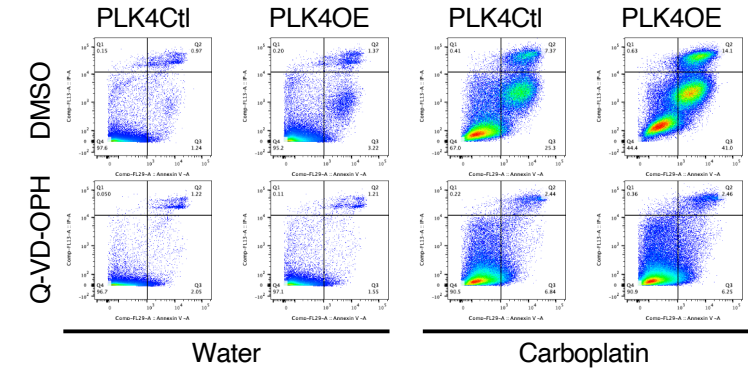

4C

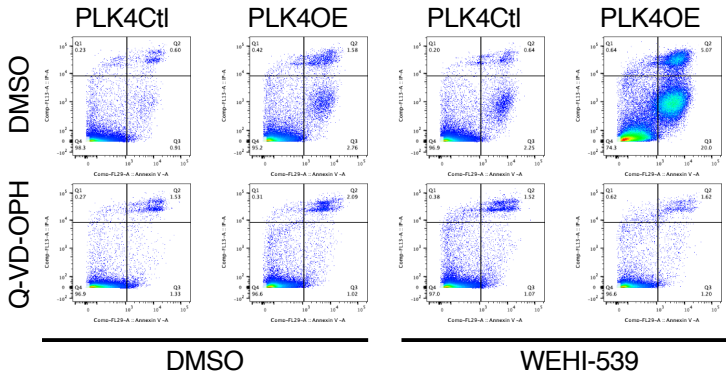

4H

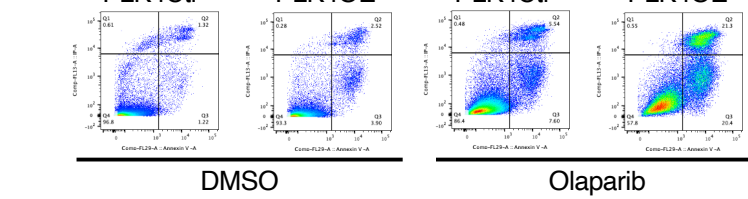

S4D

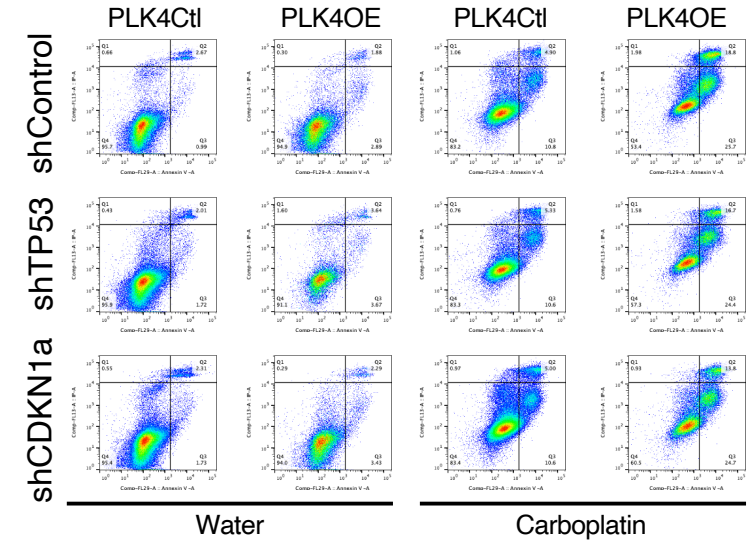

S4H

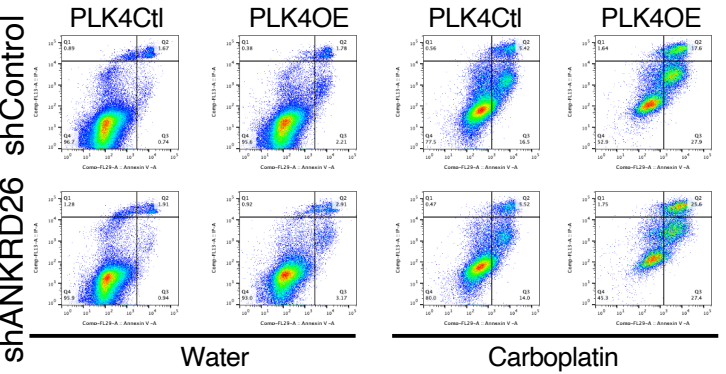

S6D

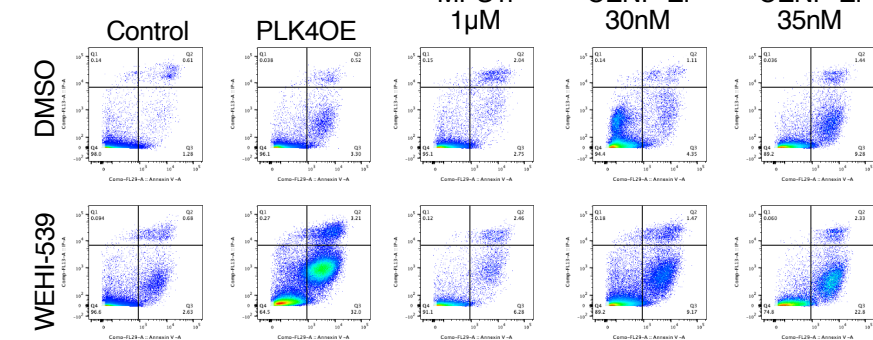

S6H

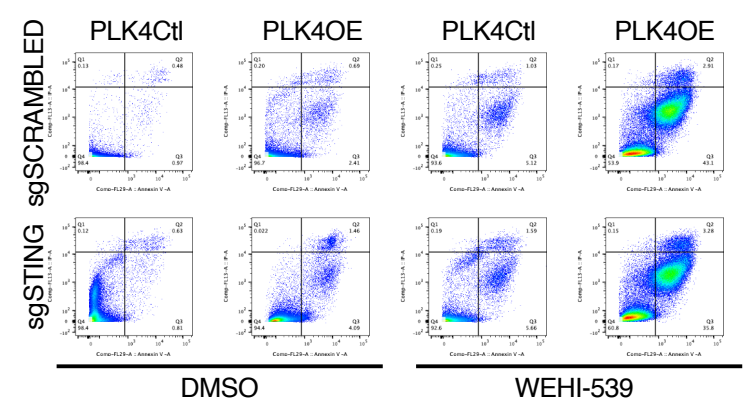

Supplement: S1 Appendix — (PDF) [file pbio.3002759.s017.pdf]
