# Supplementary material for: Centrosome amplification primes ovarian cancer cells for apoptosis and potentiates the response to chemotherapy
Source: PLoS Biol. 2024 Sep 5;22(9):e3002759. doi: 10.1371/journal.pbio.3002759 (PMC11441705; doi:10.1371/journal.pbio.3002759)
Supplement: S1 Table — (PDF) [file pbio.3002759.s013.pdf]

S1 Table. Tumor samples from Institut Curie

**Ovarian healthy samples**	**19 samples**
**Ovarian tumor samples (1990-2012):**	**100 samples**
Primary tumors (samples after surgery and before chemotherapy)	100%
**Age at diagnosis (years):**	
Median	60
Range	31-87
**Grades:**	
Low	3 (3%)
High	97 (97%)
**FIGO stages:**	
I	10(10%)
II	8 (8%)
III	59(59%)
IV	12 (12%)
NA	11 (11%)
**Histological types:**	
Serous	90 (90%)
Endometrioid	3 (3%)
Mucinous	4 (4%)
Clear cells	3 (3%)
**Treatment**	
surgery first then adjuvant chemotherapy	91 (91%)
NA	9 (9%)
**Debulking**	
Full	32 (32%)
Partial	57 (57%)
NA	11 (11%)
**Drug administered**	
Taxane derivatives+platinum salts	67 (67%)
Taxane derivatives without platinum salts	1 (1%)
Platinum salts without Taxane derivatives	16 (16%)
NA	9 (9%)
**Nb of tumors with genomic data (Affymetrix Cytoscan Array)**	81 (81%)
