# Supplementary material for: Centrosome amplification primes ovarian cancer cells for apoptosis and potentiates the response to chemotherapy
Source: PLoS Biol. 2024 Sep 5;22(9):e3002759. doi: 10.1371/journal.pbio.3002759 (PMC11441705; doi:10.1371/journal.pbio.3002759)
Supplement: S2 Table — (PDF) [file pbio.3002759.s014.pdf]

S2 Table. List of plasmids

Plasmid	Origin
pMD2.G	Addgene 12259, gift from Dider Trono
psPAX2	Addgene 12260, gift from Didier Trono
Lenti Tet-ON Myc-hPLK4 GFP selection	Gift from Andrew Holland
Lenti Tet-ON Myc-hPLK4 Blasticidin selection	Built from Lenti Tet-ON Myc-hPLK4 GFP selection, using Gibson cloning to replace the GFP selection cassette by a Blasticidin selection cassette
pENTR Age hsSAS-6 ST	Addgene 46382, gift from Pierre Gonczy
Lenti Tet-ON Myc-hSAS6dKEN	Built from Lenti Tet-ON Myc-hPLK4 GFP selection, using Gibson cloning to replace hPLK4 sequence by hSAS6 sequence from pENTR Age hsSAS6 ST. Q5 mutagenesis was then used to introduce K589A, E590A, N591A mutation.
pSMPUW-IRIS-Neo-H2B-RFP	Gift from Daniele Facchinetti
pBOB-EF1-FastFUCCI-Puro	Addgene 86849, gift from Kevin Brindle and Duncan Jodrell
L304-EGFP-Tubulin-WT	Addgene 64060
pLKo.1-puro shRNA Control	Sigma-Aldrich SHC016-1EA
pLKo.1-puro shRNA TP53	Gift from Daniele Facchinetti
pLKo.1-puro shRNA CDKN1a	Horizon Discovery RHS3979- 200795839
pLKo.1-puro shRNA ANKRD26 1	Horizon Discovery RHS3979-201867496
pLKo.1-puro shRNA ANKRD26 2	Horizon Discovery RHS3979- 201865755
sgRNA CRISPR Control	Gift from Nicolas Manel
sgRNA CRISPR STING	Gift from Nicolas Manel
