# Supplementary material for: Centrosome amplification primes ovarian cancer cells for apoptosis and potentiates the response to chemotherapy
Source: PLoS Biol. 2024 Sep 5;22(9):e3002759. doi: 10.1371/journal.pbio.3002759 (PMC11441705; doi:10.1371/journal.pbio.3002759)
Supplement: S3 Table — (PDF) [file pbio.3002759.s015.pdf]

S3 Table. List of chemicals

Chemical	Origin	Stock dilution
Doxycyline	Sigma-Aldrich D3447	10 mg/mL in DMSO
Puromycine dichlorhydrate	ThermoFisher Scientific A1113803	10 mg/mL in 20mM HEPES
Carboplatin	Selleck chemicals S1215	10mM in Water
Paclitaxel	Sigma-Aldrich T7402	10mM in DMSO
A1210477	MedChem Express HY-12468	10mM in DMSO
WEHI-539 hydrochloride	MedChem Express HY-15607A	5mM in DMSO
Navitoclax	Selleck chemicals S1001	10mM in DMSO
Venetoclax	MedChem Express HY-15531	10mM in DMSO
AZ3146	Selleck chemicals S2731	10mM in DMSO
GSK923295	Selleck chemicals S7090	10mM in DMSO
Q-VD-Oph	MedChem Express HY-12305	20mM in DMSO
DMSO	ThermoFisher Scientific 022914.M1	
