# Supplementary material for: Centrosome amplification primes ovarian cancer cells for apoptosis and potentiates the response to chemotherapy
Source: PLoS Biol. 2024 Sep 5;22(9):e3002759. doi: 10.1371/journal.pbio.3002759 (PMC11441705; doi:10.1371/journal.pbio.3002759)
Supplement: S4 Table — (PDF) [file pbio.3002759.s016.pdf]

S4 Table. List of antibodies

Antibody	Origin	Species
CEP192	Home-made	Guinea pig
Pericentrin	Abcam 28144	Mouse
Pericentrin	Abcam 4448	Rabbit
Centrin 3	Home-made	Rabbit
Centromere	Antibodies Incorporated 15-234	Human
Cytochrome C	ThermoFisher Scientific 15808698	Mouse
gamma-H2AX (S139)	Abcam 22551	Mouse
Rad51	Abcam 133534	Rabbit
53BP1	Millipoare MAB3802	Mouse
FANCD2	Novus Biologicals 100-182	Rabbit
Caspase3	Cell Signaling Technology 14220	Rabbit
Cleaved Caspase 3	Cell Signaling Technology 9661	Rabbit
pChk1 (Ser317)	Cell Signaling Technology 2344	Rabbit
Chk1	Santa Cruz 8408	Mouse
pChk2 (Thr68)	Cell Signaling Technology 2661	Rabbit
Chk2	Cell Signaling Technology 2662	Rabbit
pp53 (Ser15)	Cell Signaling Technology 9284	Rabbit
p53	Santa Cruz 126	Mouse
p21	Millipore OP64-100UG	Mouse
PUMA	Santa Cruz 374223	Mouse
Caspase2	Millipore MAB3507	Rabbit
MDM2	ThermoFisher Scientific MA1-113	Mouse
ANKRD26	GeneTex GTX128255	Rabbit
pSTING	Cell Signaling Technology 19781	Rabbit
STING	Cell Signaling Technology 13647	Rabbit
GAPDH	Sigma-Aldrich G9545-100UL	Rabbit
HRP-coupled anti-rabbit	ThermoFisher G21234	Goat
HRP-coupled anti-mouse	Jackson ImmunoResearch 115-035-003	Goat
anti-mouse IgG (H+L) Highly Cross-Adsorbed Secondary Antibody, Alexa Fluor 647	ThermoFisher Scientific A-21245	Goat
anti-rabbit IgG (H+L) Highly Cross-Adsorbed Secondary Antibody, Alexa Fluor 647	ThermoFisher Scientific A-21245	Goat
anti-guinea pig IgG (H+L) Highly Cross-Adsorbed Secondary Antibody, Alexa Fluor 647	ThermoFisher Scientific A-21450	Goat
anti-mouse IgG (H+L) Cross-Adsorbed Second-	ThermoFisher Scientific A-11003	Goat

ary Antibody, Alexa Fluor 546		
anti-rabbit IgG (H+L) Highly Cross-Adsorbed Secondary Antibody, Alexa Fluor 546	Thermo Fisher Scientific A-11035	Goat
anti-guinea pig IgG (H+L) Highly Cross-Adsorbed Secondary Antibody, Alexa Fluor 568	ThermoFisher Scientific A-11075	Goat
anti-mouse IgG (H+L) Highly Cross-Adsorbed Secondary Antibody, Alexa Fluor 488	ThermoFisher Scientific A-11029	Goat
