# Supplementary material for: Centrosome amplification primes ovarian cancer cells for apoptosis and potentiates the response to chemotherapy
Source: PLoS Biol. 2024 Sep 5;22(9):e3002759. doi: 10.1371/journal.pbio.3002759 (PMC11441705; doi:10.1371/journal.pbio.3002759)

Fig 3A

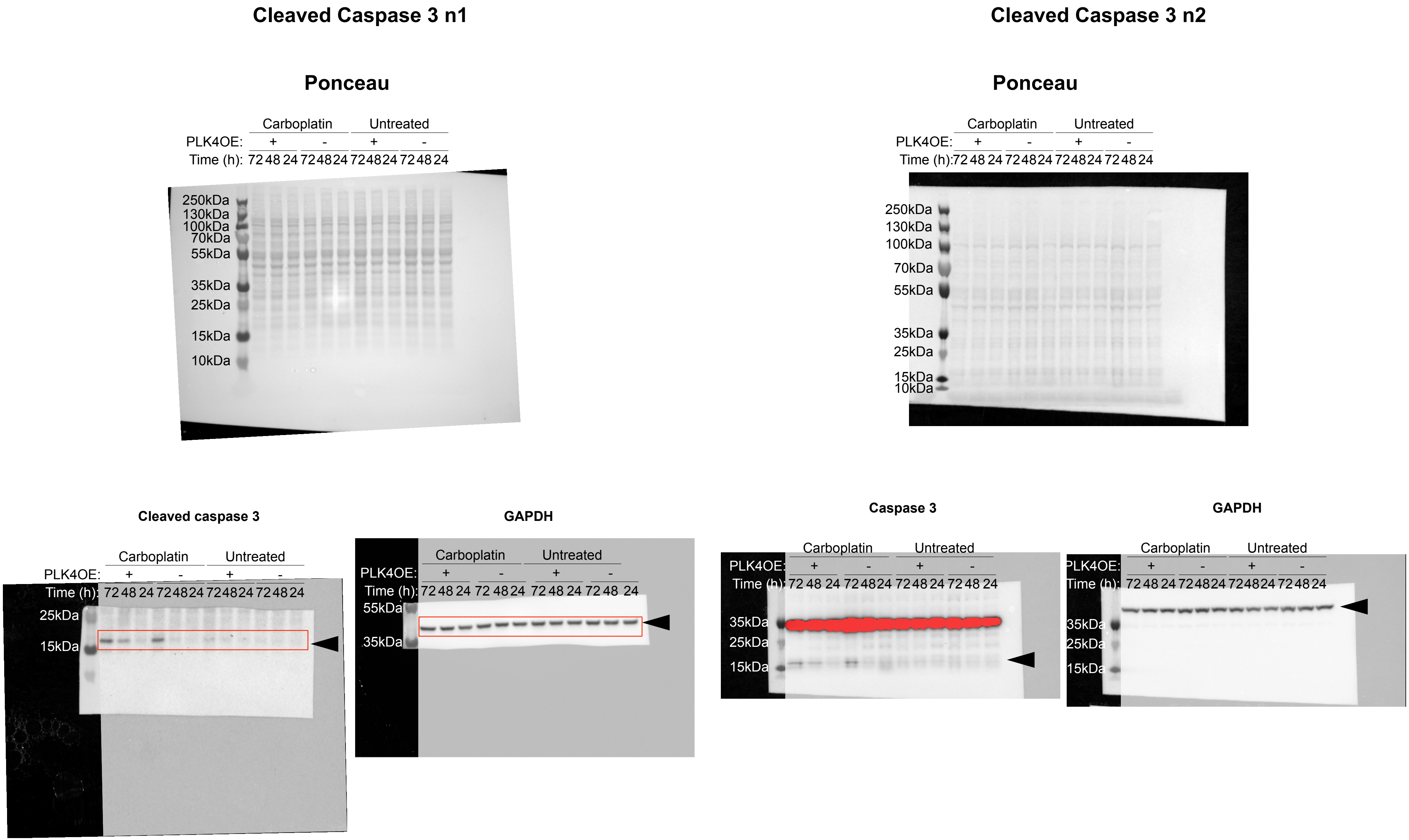
Cleaved Caspase 3 n1
Ponceau
Carboplatin
Untreated
PLK4OE: + - + -
Time (h): 72 48 24 72 48 24 72 48 24 72 48 24
250kDa
130kDa
100kDa
70kDa
55kDa
35kDa
25kDa
15kDa
10kDa
Cleaved Caspase 3 n2
Ponceau
Carboplatin
Untreated
PLK4OE: + - + -
Time (h): 72 48 24 72 48 24 72 48 24 72 48 24
250kDa
130kDa
100kDa
70kDa
55kDa
35kDa
25kDa
15kDa
10kDa
Cleaved caspase 3
Carboplatin
Untreated
PLK4OE: + - + -
Time (h): 72 48 24 72 48 24 72 48 24 72 48 24
25kDa
15kDa
GAPDH
Carboplatin
Untreated
PLK4OE: + - + -
Time (h): 72 48 24 72 48 24 72 48 24 72 48 24
55kDa
35kDa
Caspase 3
Carboplatin
Untreated
PLK4OE: + - + -
Time (h): 72 48 24 72 48 24 72 48 24 72 48 24
35kDa
25kDa
15kDa
GAPDH
Carboplatin
Untreated
PLK4OE: + - + -
Time (h): 72 48 24 72 48 24 72 48 24 72 48 24
35kDa
25kDa
15kDa

Fig S3G

Chk1 and pChk1 n1

Chk1 ponceau

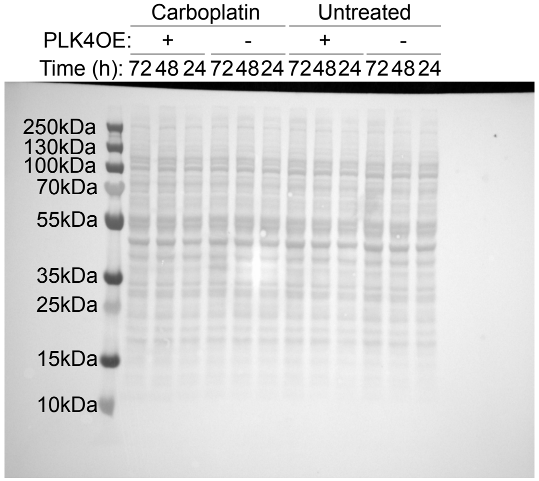

pChk1 ponceau

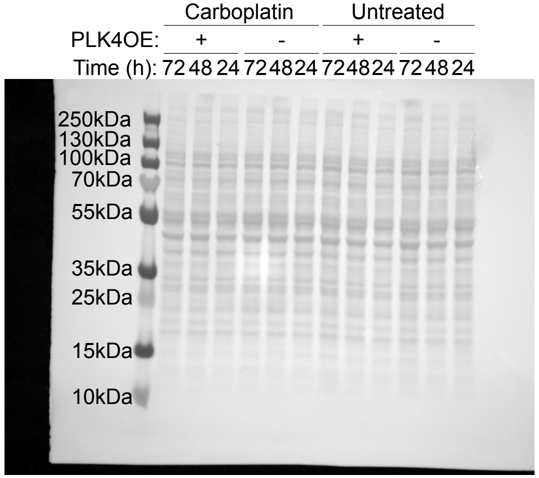

Chk1 and pChk1 n2

Ponceau,Same membrane for pChk1 and Chk1, stripped in between

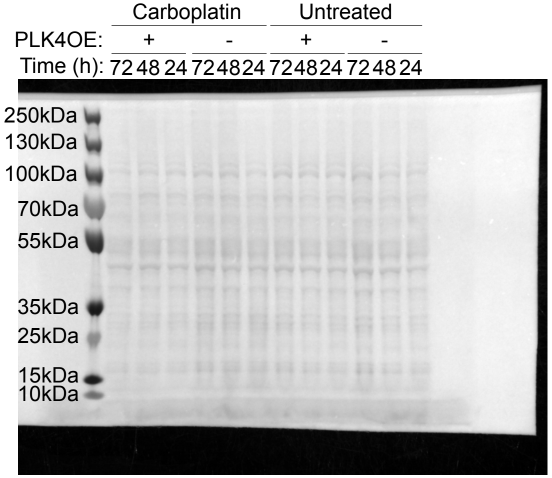

Chk1

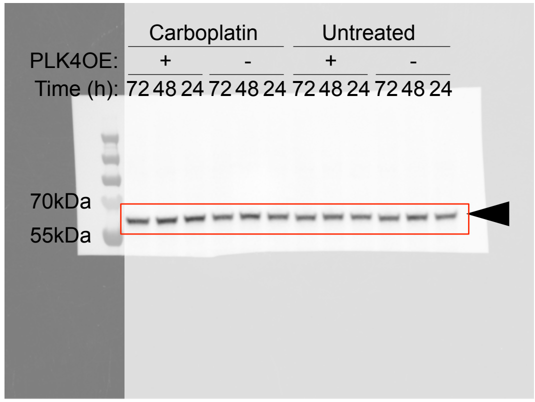

pChk1

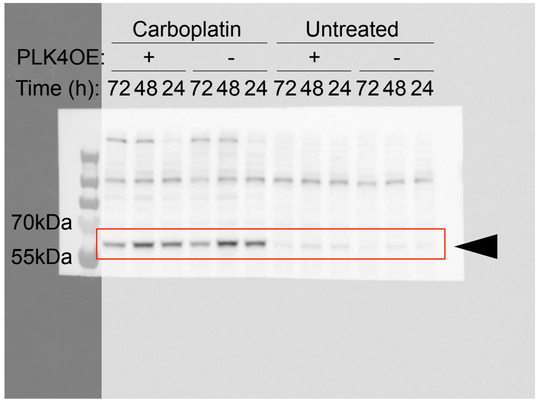

Chk1

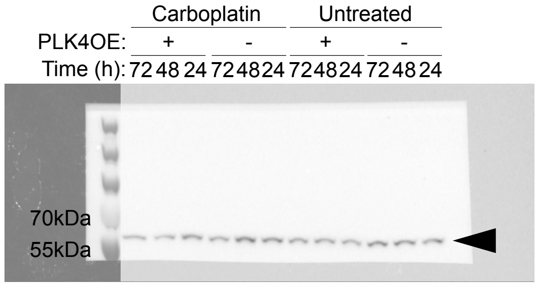

pChk1

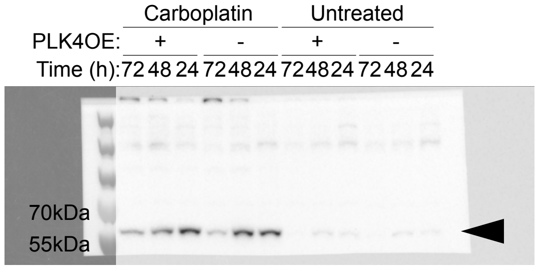

Fig S3G

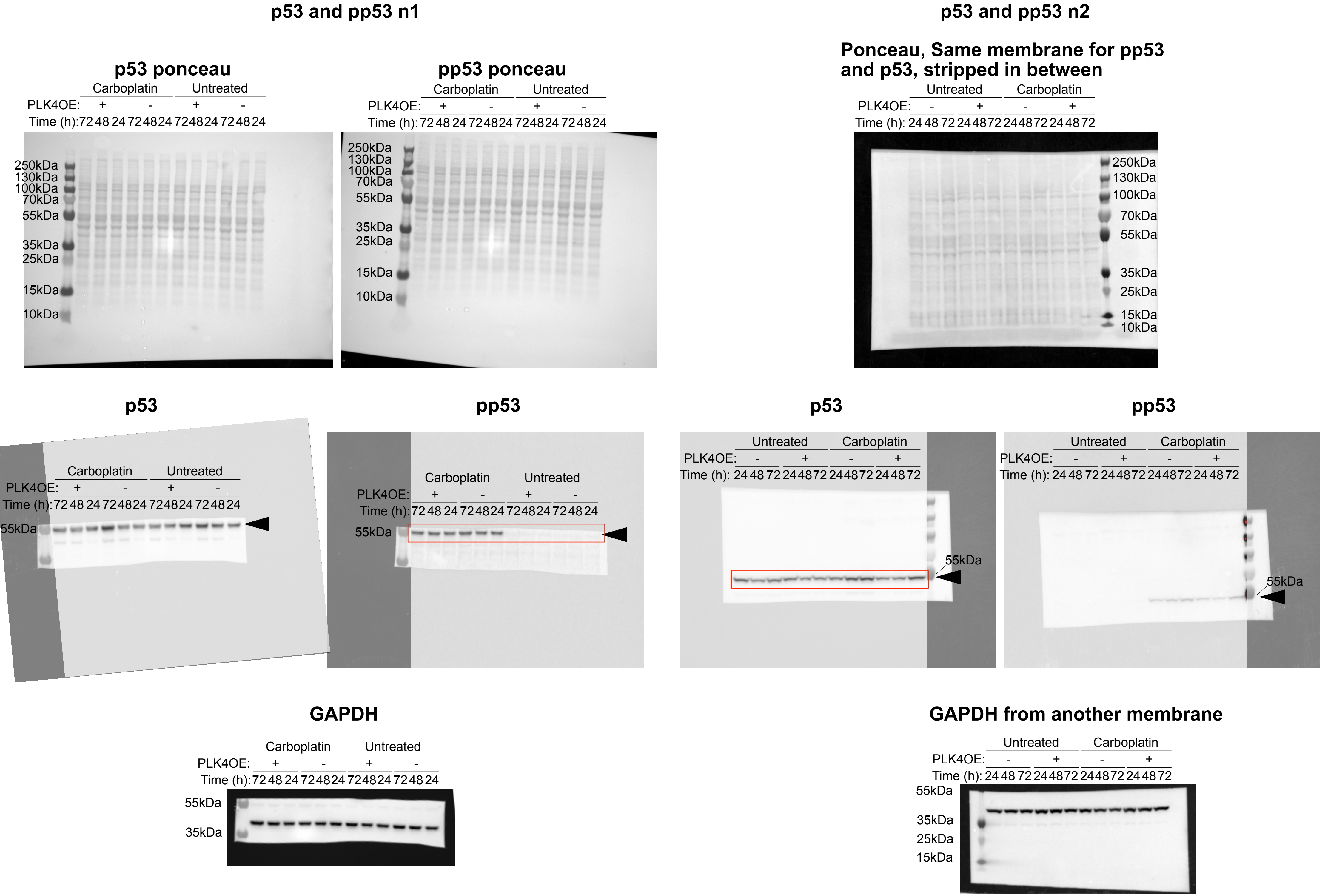
p53 and pp53 n1
p53 ponceau
Carboplatin Untreated
PLK4OE: + - + -
Time (h): 72 48 24 72 48 24 72 48 24 72 48 24
250kDa
130kDa
100kDa
70kDa
55kDa
35kDa
25kDa
15kDa
10kDa
pp53 ponceau
Carboplatin Untreated
PLK4OE: + - + -
Time (h): 72 48 24 72 48 24 72 48 24 72 48 24
250kDa
130kDa
100kDa
70kDa
55kDa
35kDa
25kDa
15kDa
10kDa
p53 and pp53 n2
Ponceau, Same membrane for pp53 and p53, stripped in between
Untreated Carboplatin
PLK4OE: - + - +
Time (h): 24 48 72 24 48 72 24 48 72 24 48 72
250kDa
130kDa
100kDa
70kDa
55kDa
35kDa
25kDa
15kDa
10kDa
p53
Carboplatin Untreated
PLK4OE: + - + -
Time (h): 72 48 24 72 48 24 72 48 24 72 48 24
55kDa
pp53
Carboplatin Untreated
PLK4OE: + - + -
Time (h): 72 48 24 72 48 24 72 48 24 72 48 24
55kDa
p53
Untreated Carboplatin
PLK4OE: - + - +
Time (h): 24 48 72 24 48 72 24 48 72 24 48 72
55kDa
pp53
Untreated Carboplatin
PLK4OE: - + - +
Time (h): 24 48 72 24 48 72 24 48 72 24 48 72
55kDa
GAPDH
Carboplatin Untreated
PLK4OE: + - + -
Time (h): 72 48 24 72 48 24 72 48 24 72 48 24
55kDa
35kDa
GAPDH from another membrane
Untreated Carboplatin
PLK4OE: - + - +
Time (h): 24 48 72 24 48 72 24 48 72 24 48 72
55kDa
35kDa
25kDa
15kDa

Fig S4A

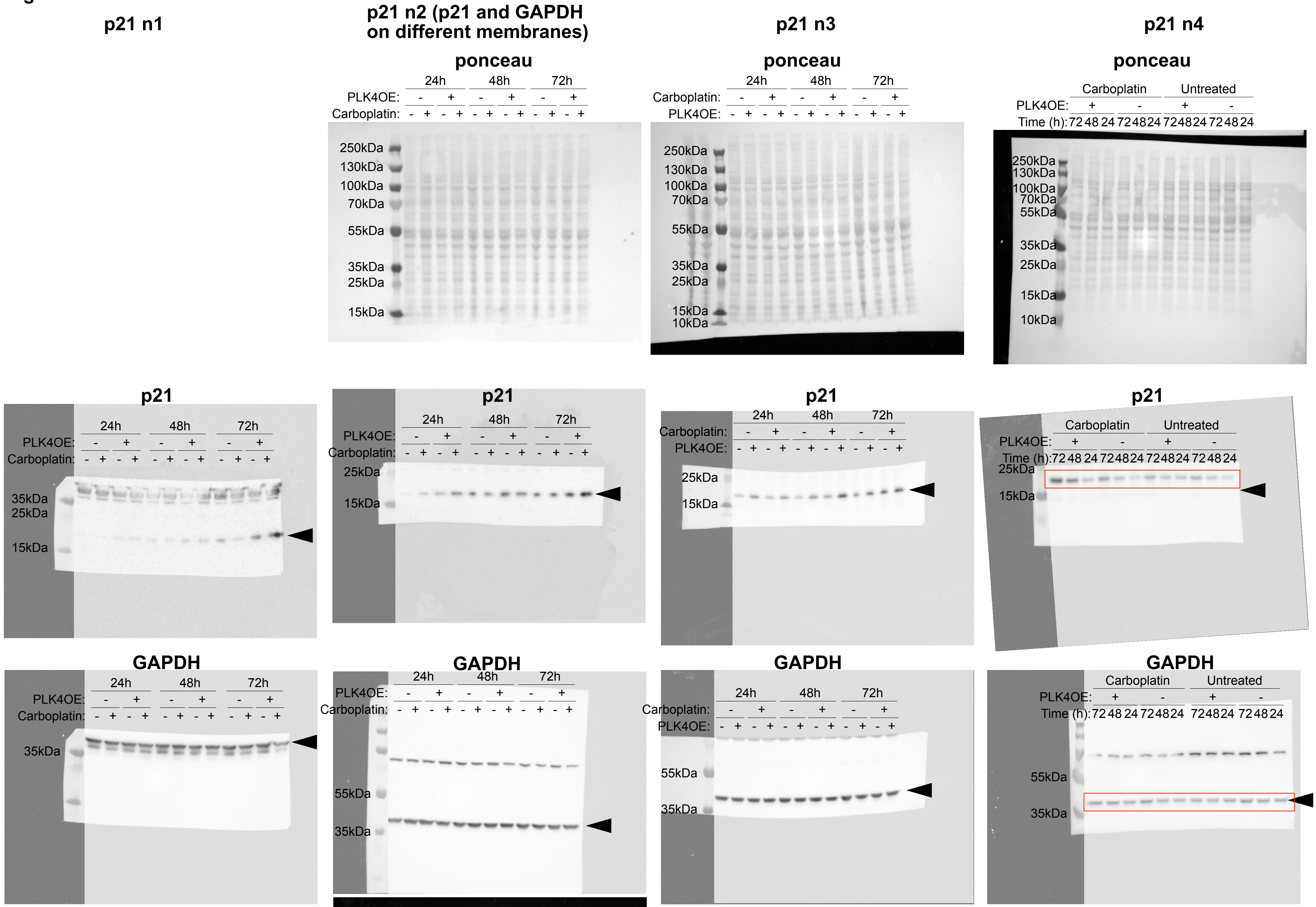
p21 n1
p21 n2 (p21 and GAPDH on different membranes)
p21 n3
p21 n4
ponceau
24h 48h 72h
PLK4OE: - + - + - +
Carboplatin: - + - + - + - + - + - +
250kDa
130kDa
100kDa
70kDa
55kDa
35kDa
25kDa
15kDa
ponceau
24h 48h 72h
Carboplatin: - + - + - +
PLK4OE: - + - + - + - + - + - +
250kDa
130kDa
100kDa
70kDa
55kDa
35kDa
25kDa
15kDa
10kDa
ponceau
Carboplatin Untreated
PLK4OE: + - + -
Time (h): 72 48 24 72 48 24 72 48 24 72 48 24
250kDa
130kDa
100kDa
70kDa
55kDa
35kDa
25kDa
15kDa
10kDa
p21
24h 48h 72h
PLK4OE: - + - + - +
Carboplatin: - + - + - + - + - + - +
35kDa
25kDa
15kDa
p21
24h 48h 72h
PLK4OE: - + - + - +
Carboplatin: - + - + - + - + - + - +
25kDa
15kDa
p21
24h 48h 72h
Carboplatin: - + - + - +
PLK4OE: - + - + - + - + - + - +
25kDa
15kDa
p21
Carboplatin Untreated
PLK4OE: + - + -
Time (h): 72 48 24 72 48 24 72 48 24 72 48 24
25kDa
15kDa
GAPDH
24h 48h 72h
PLK4OE: - + - + - +
Carboplatin: - + - + - + - + - + - +
35kDa
GAPDH
24h 48h 72h
PLK4OE: - + - + - +
Carboplatin: - + - + - + - + - + - +
55kDa
35kDa
GAPDH
24h 48h 72h
Carboplatin: - + - + - +
PLK4OE: - + - + - + - + - + - +
55kDa
35kDa
GAPDH
Carboplatin Untreated
PLK4OE: + - + -
Time (h): 72 48 24 72 48 24 72 48 24 72 48 24
55kDa
35kDa

Fig S4A

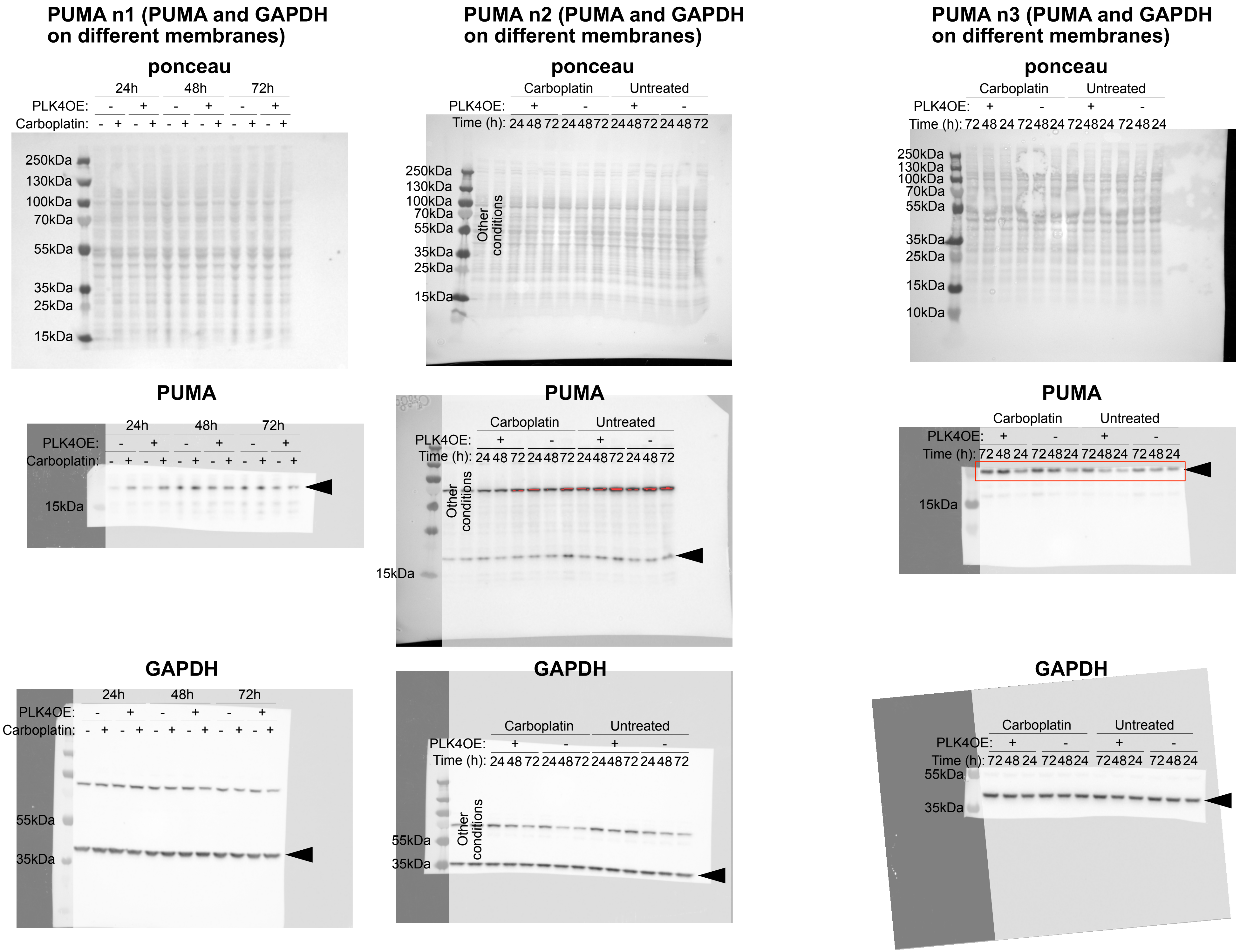
PUMA n1 (PUMA and GAPDH on different membranes)
PUMA n2 (PUMA and GAPDH on different membranes)
PUMA n3 (PUMA and GAPDH on different membranes)
ponceau
PUMA
GAPDH
24h
48h
72h
PLK4OE:
Carboplatin:
Carboplatin
Untreated
Time (h):
Other conditions
250kDa
130kDa
100kDa
70kDa
55kDa
35kDa
25kDa
15kDa
10kDa

Fig S4B p53 quantification

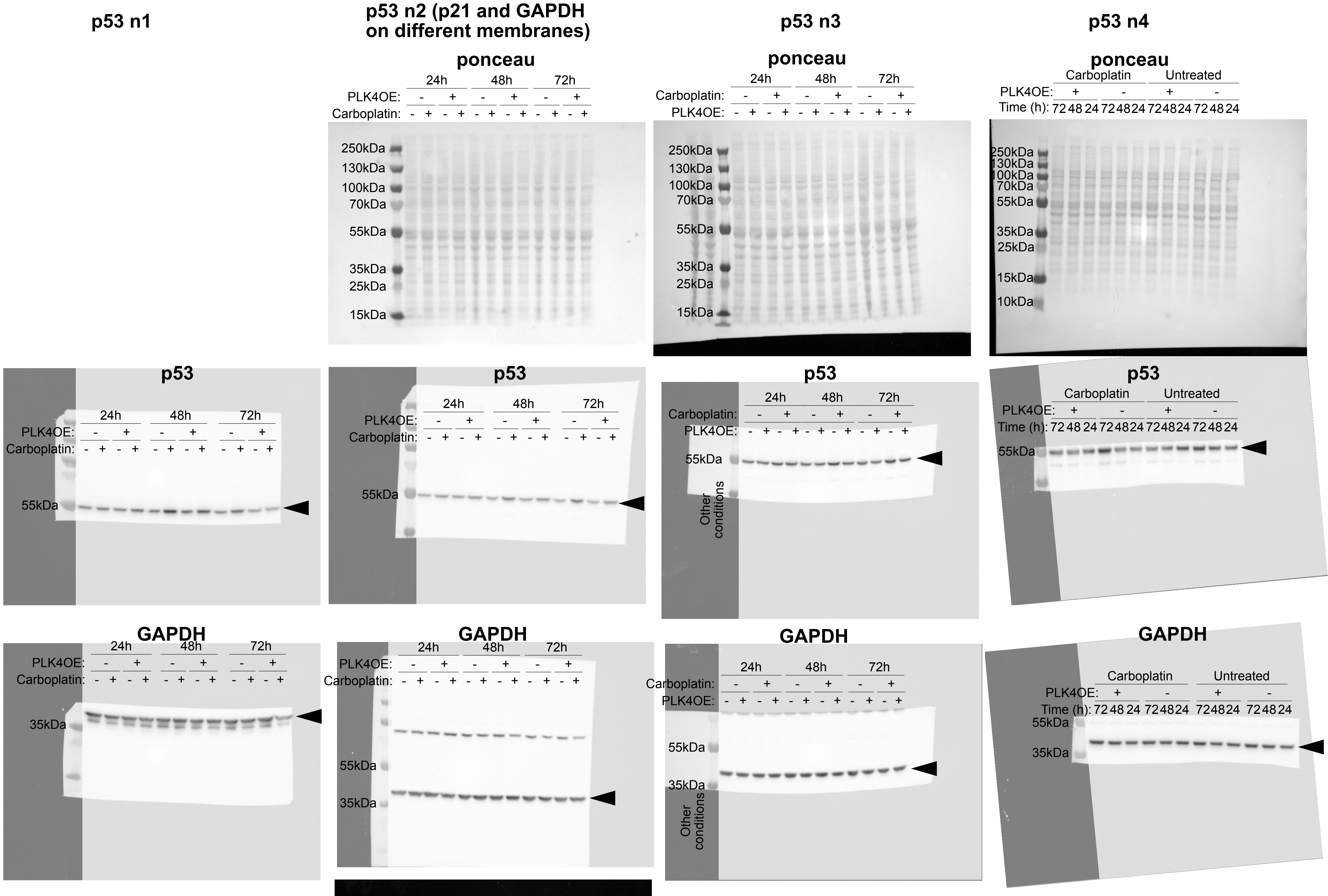

Fig S4C

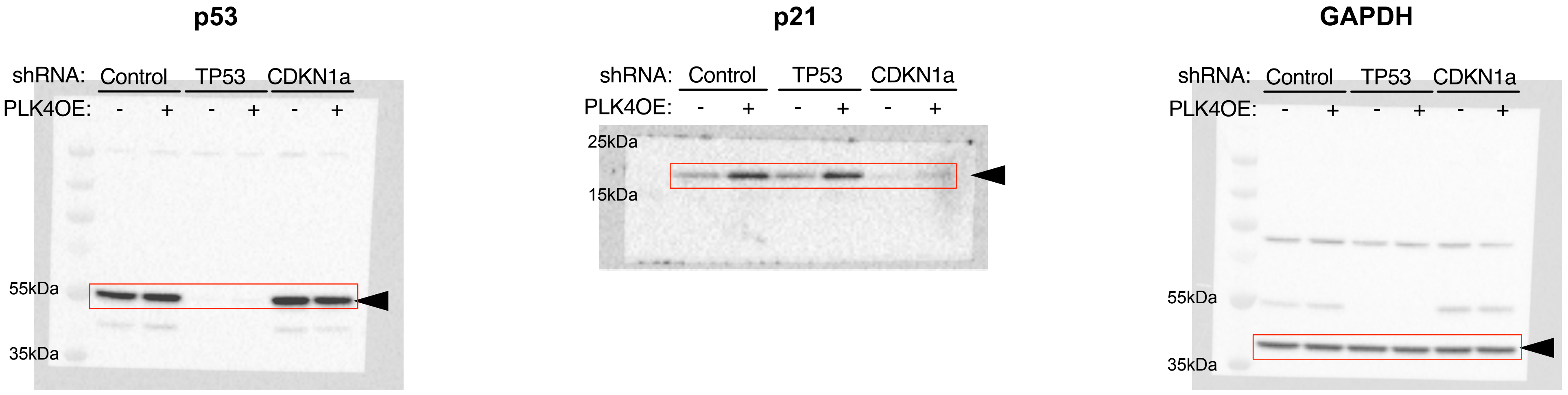
p53
shRNA: Control TP53 CDKN1a
PLK4OE: - + - + - +
55kDa
35kDa
p21
shRNA: Control TP53 CDKN1a
PLK4OE: - + - + - +
25kDa
15kDa
GAPDH
shRNA: Control TP53 CDKN1a
PLK4OE: - + - + - +
55kDa
35kDa

Fig S4E

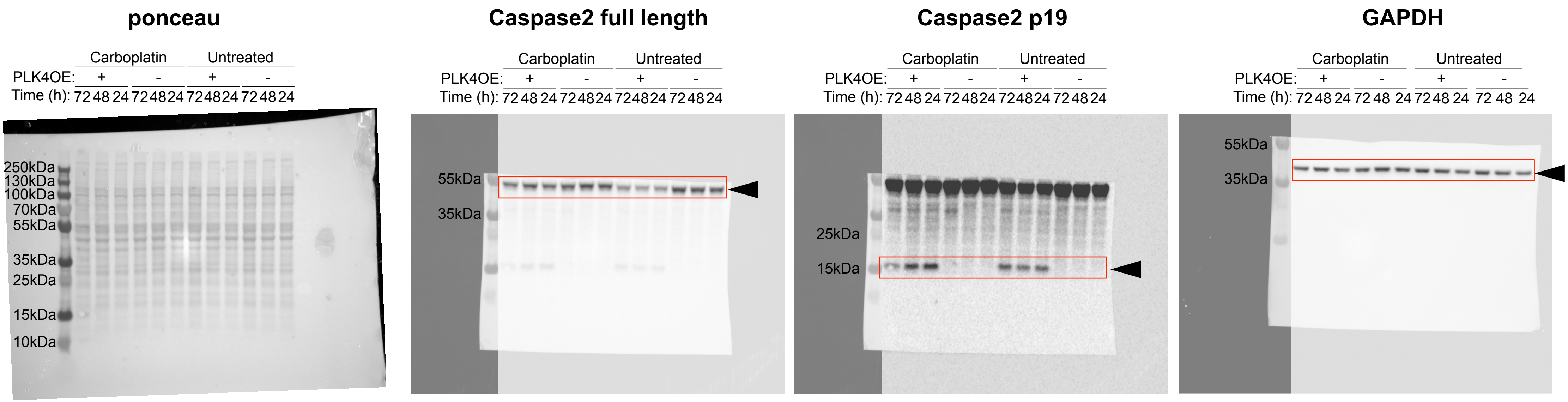
ponceau
Caspase2 full length
Caspase2 p19
GAPDH
Carboplatin
Untreated
PLK4OE: + - + -
Time (h): 72 48 24 72 48 24 72 48 24 72 48 24
250kDa
130kDa
100kDa
70kDa
55kDa
35kDa
25kDa
15kDa
10kDa

Fig S4E

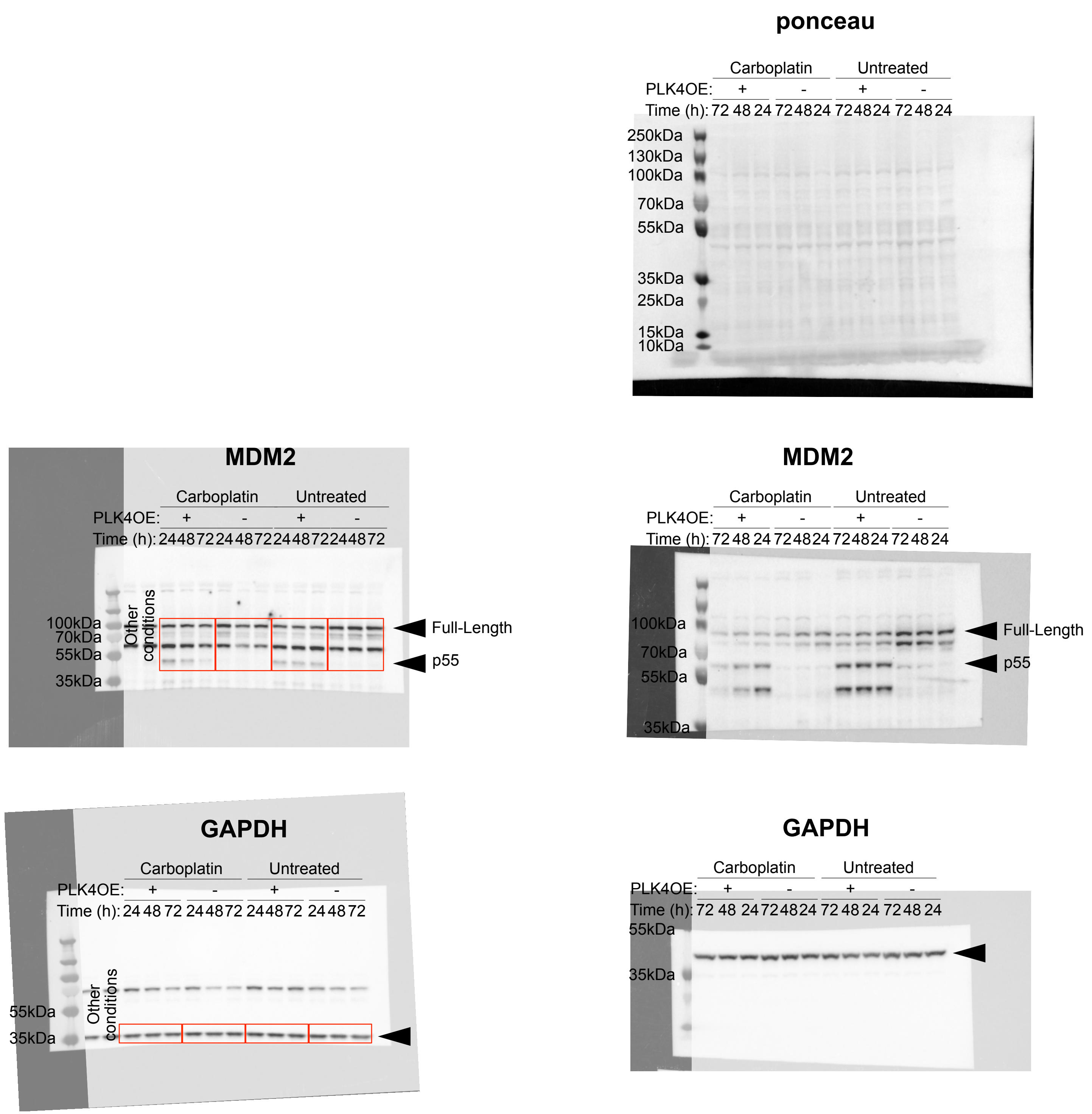

ponceau
Carboplatin
Untreated
PLK4OE: + - + -
Time (h): 72 48 24 72 48 24 72 48 24 72 48 24
250kDa
130kDa
100kDa
70kDa
55kDa
35kDa
25kDa
15kDa
10kDa
MDM2
Carboplatin
Untreated
PLK4OE: + - + -
Time (h): 24 48 72 24 48 72 24 48 72 24 48 72
100kDa
70kDa
55kDa
35kDa
Other conditions
Full-Length
p55
MDM2
Carboplatin
Untreated
PLK4OE: + - + -
Time (h): 72 48 24 72 48 24 72 48 24 72 48 24
100kDa
70kDa
55kDa
35kDa
Full-Length
p55
GAPDH
Carboplatin
Untreated
PLK4OE: + - + -
Time (h): 24 48 72 24 48 72 24 48 72 24 48 72
55kDa
35kDa
Other conditions
GAPDH
Carboplatin
Untreated
PLK4OE: + - + -
Time (h): 72 48 24 72 48 24 72 48 24 72 48 24
55kDa
35kDa

Fig S4F

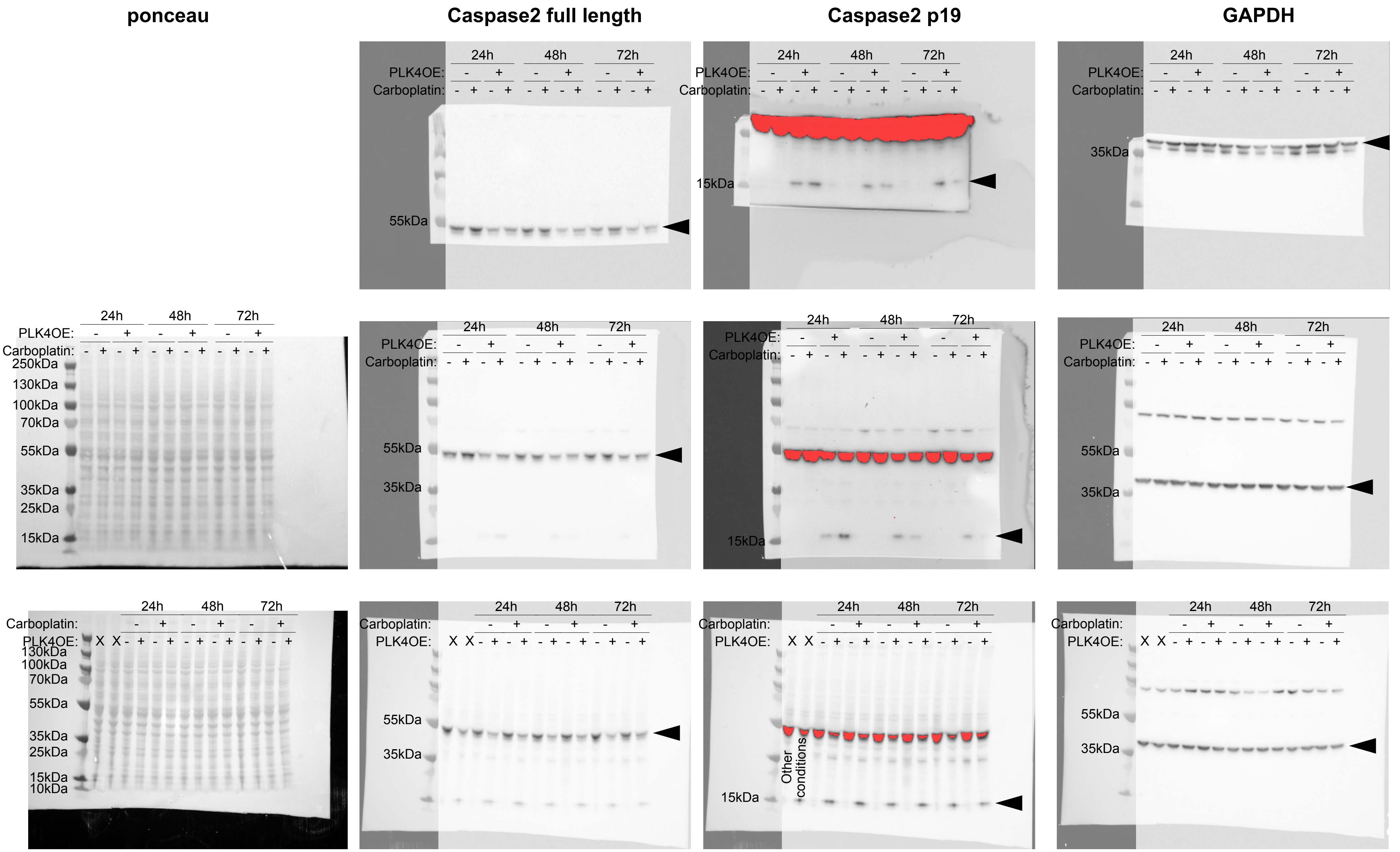

ponceau
Caspase2 full length
Caspase2 p19
GAPDH
24h
48h
72h
PLK4OE:
Carboplatin:
250kDa
130kDa
100kDa
70kDa
55kDa
35kDa
25kDa
15kDa
10kDa
X
Other conditions

Fig S4G

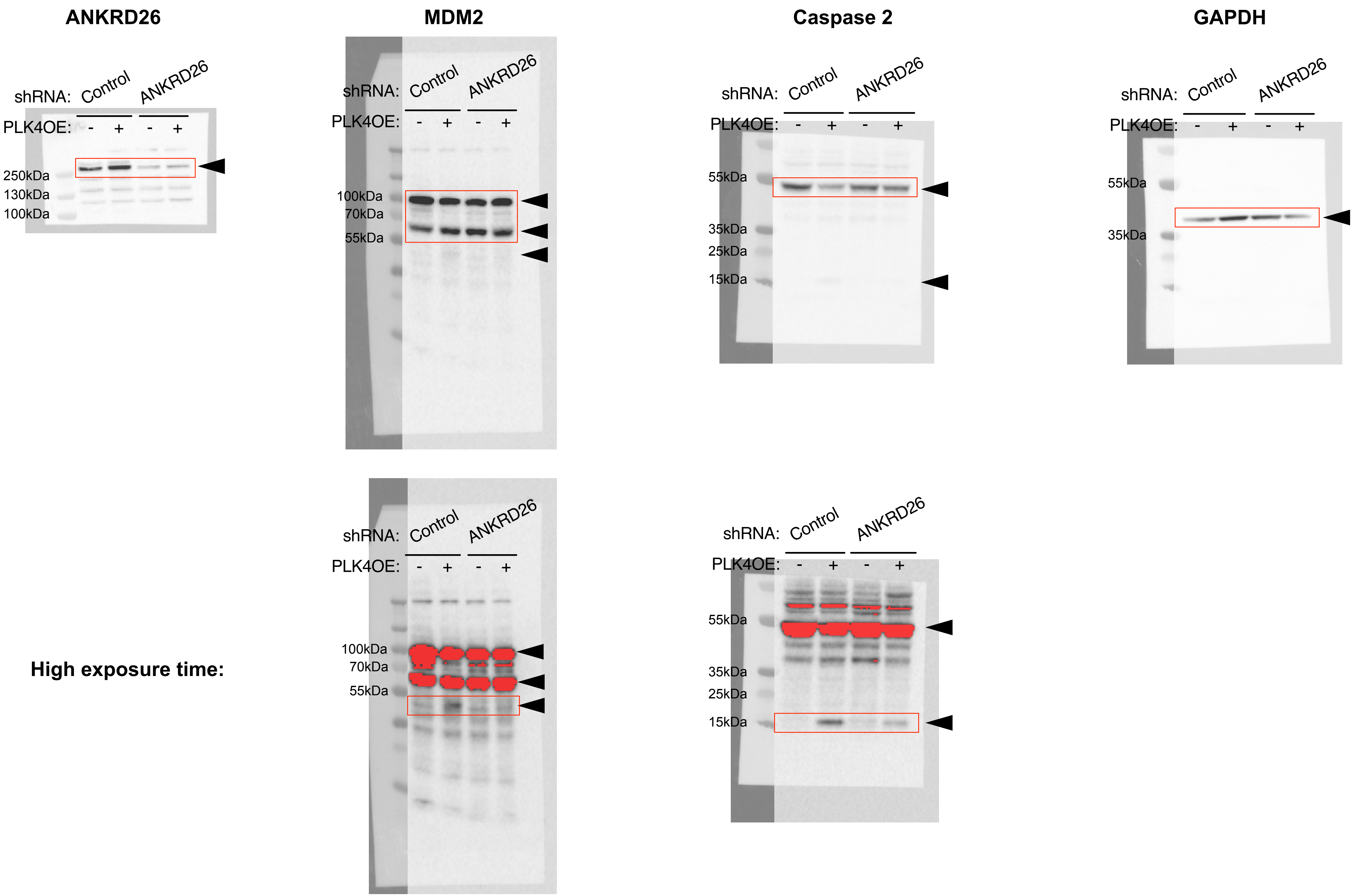
ANKRD26
MDM2
Caspase 2
GAPDH
shRNA: Control ANKRD26
PLK4OE: - + - +
250kDa
130kDa
100kDa
70kDa
55kDa
35kDa
25kDa
15kDa
High exposure time:

Fig S6F

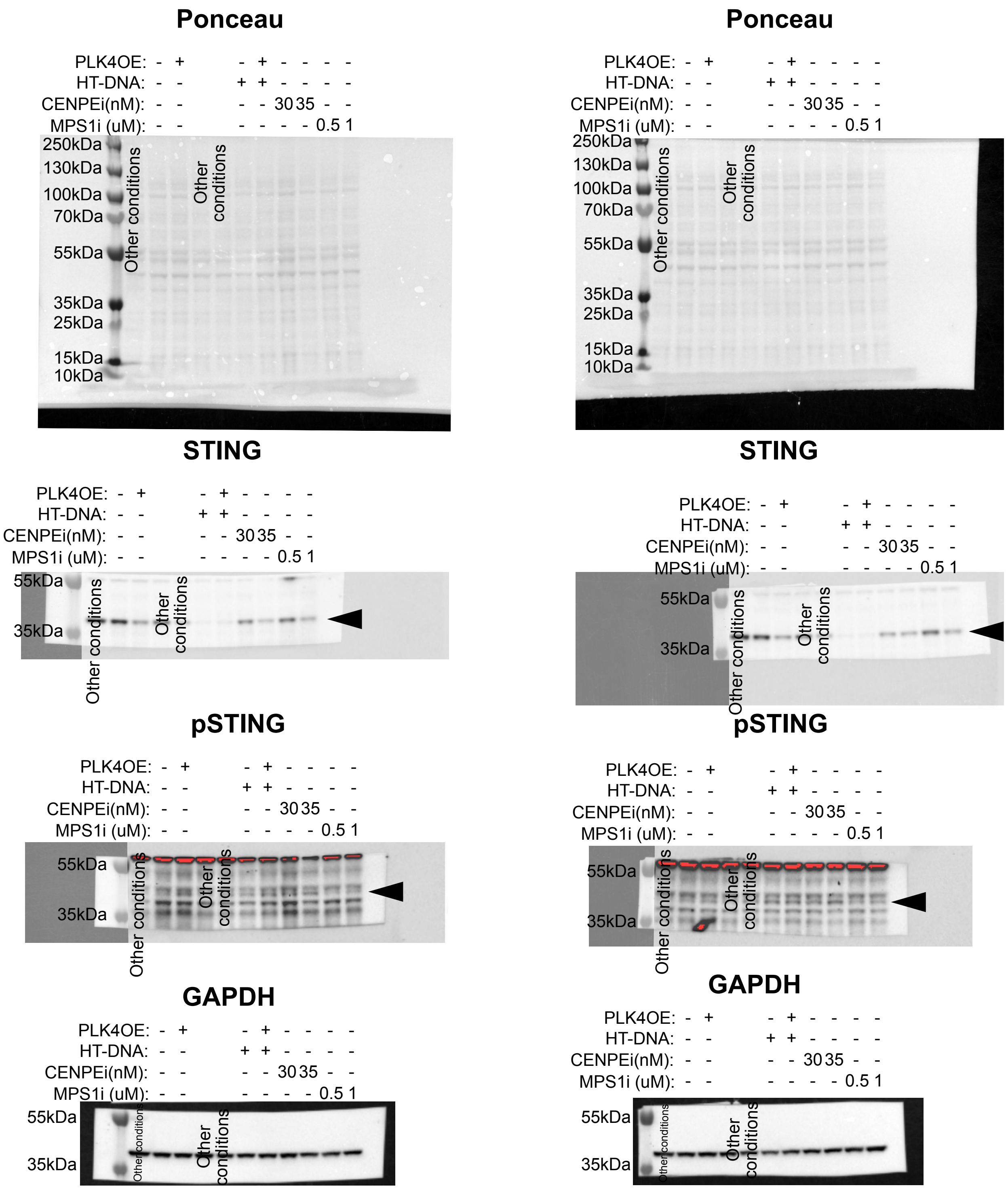
Ponceau
PLK4OE: - + - + - - - -
HT-DNA: - - + + - - - -
CENPEi(nM): - - - - 30 35 - -
MPS1i (uM): - - - - - - 0.5 1
250kDa
130kDa
100kDa
70kDa
55kDa
35kDa
25kDa
15kDa
10kDa
Other conditions
Other conditions
STING
55kDa
35kDa
pSTING
GAPDH

Fig S6G

Ponceau

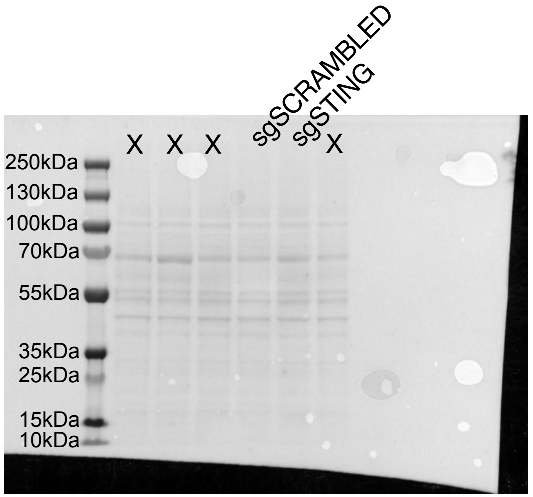

STING

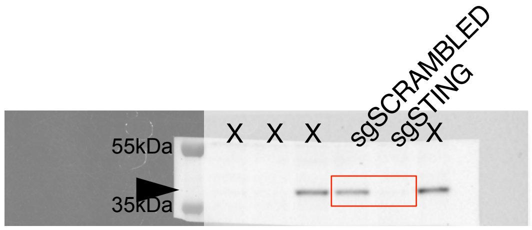

GAPDH

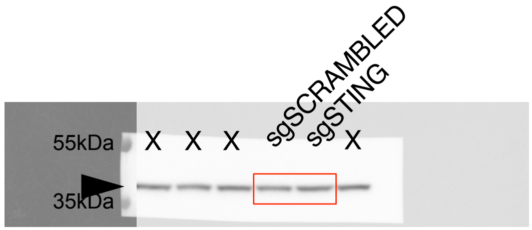

Supplement: S1 Raw Images — (PDF) [file pbio.3002759.s018.pdf]
