# Supplementary material for: Centrosome amplification primes ovarian cancer cells for apoptosis and potentiates the response to chemotherapy
Source: PLoS Biol. 2024 Sep 5;22(9):e3002759. doi: 10.1371/journal.pbio.3002759 (PMC11441705; doi:10.1371/journal.pbio.3002759)
Supplement: Attachment — Submitted filename: Edwards et al, Response to reviewers R2.pdf [file pbio.3002759.s034.pdf]

REVIEWS:

Reviewer #1: The revised manuscript from Edwards et al is improved, though some concerns remain. Some of the previous critiques have been addressed, including acknowledging that it remains unclear how the priming observed in a population in which ~80% of cells have centrosome amplification would operate in patient tumors with ≤3% centrosome amplification. However, multiple concerns raised have been only partially addressed, and some conclusions presented are too strong based on the available evidence. Additional modifications are warranted prior to publication.

1. The last paragraph of the Introduction and the Results on page 7 accurately describe the significant increase in multipolar spindles in PLK4OE cells as contributing to the increase in cell death in response to paclitaxel+carboplatin. However, the summary and discussion continue to emphasize that PLK4 overexpression induces cell death "independently" of mitotic defects and the revised title is confusing. In keeping with their wording in the last paragraph of the Introduction, changing "besides" to "beyond" in the title would increase clarity (ie "Centrosome amplification primes for apoptosis beyond promoting multipolar divisions…). In the abstract, rather than centrosome amplification acting "independently of" (line 34) multipolar divisions it would be more appropriate to indicate this effect is "over and above" or "in addition to" multipolar divisions.

As requested by the reviewer, we have changed the title, introduction and abstract. All the changes are highlighted in yellow.

2. The inability to visualize tubulin, as proposed in the revision plan but not accomplished in the revision, remains a significant weakness. Since the primary mitotic phenotype expected from centrosome amplification is (at least transient) multipolar spindles, it is critical to visualize tubulin to rule this out. Fluorescently tagged tubulin, which can be incorporated after transfection or viral infection, is widely used to visualize mitotic spindles and it is unclear why this approach was not used here, where it would be expected to succeed in this cell line. Instead the authors treated cells with fluorescent taxane, which unsurprisingly caused a mitotic phenotype that precluded interpretation of results. The fixed analysis of tubulin after PLK4OE alone (Fig S2) somewhat mitigates this concern, though the finding that the additional centrosomes do not efficiently form spindle poles is surprising given their ability to do so in other contexts, raising questions about the generality of these findings. Conclusions about neglible multipolar divisions and absence of mitotic errors based on timelapse analysis that does not include tubulin should be tempered based on the available evidence.

We have been reluctant to generate these cancer cell lines with GFP-tubulin, as in our hands and independently of the model system used, GFP-Tubulin can cause mitotic phenotypes or even cell death. In other words GFP-Tubulin can be highly toxic in a variety of experimental set ups. This is certainly true in flies, mice and several cell lines used in our lab. But since the reviewer strongly believes that this is an important point and that our fixed and live imaging approaches do not rule out a major contribution of multipolarity to the phenotypes described, we have generated iOVCAR8 cell lines stably expressing GFP-Tubulin delivered by lentivirus. We had to select for low-GFP-expressing cells, as the ones that expressed moderate to high GFP levels showed abnormal mitotic behaviours and very high levels of apoptosis

when in culture without any further treatment, which is not the case for iOVCAR8 cell lines without GFP-Tubulin expression. The selection was made by FACS.

After generating iPLK4OE cell lines expressing both GFP-Tubulin and H2B-mCherry constructs, we were able to characterize mitosis in cells with extra centrosomes ± carboplatin. As shown in Figure S2H, we show that multipolarity remains a minor behaviour in these cells (PLK4OE DMSO: 6.5% and PLK4OE Carboplatin: 4.0%). We reason that GFP-Tubulin expression is indeed toxic as the percentage of death in mitosis increases, but also because mitotic length is extended. We describe these finding in the text, figure legends and images provided in Fig S2H-I. Importantly, as described for the fixed images before, we see centrosomal clustering, but also centrosomes that localise to the mitotic bipolar spindles but not at spindle poles. This new cell line confirms our results that carboplatin synergizes with centrosome amplification independently of generating multipolar divisions.

3. The images for cytochrome C release in Fig 4D and 4G are still not persuasive. The enlargements in 4G are smaller than those in 3D and have more colors, resulting in images that are very difficult to discern. The low contrast between yellow and white is suboptimal.

We are sorry that the reviewer does not find these images illustrative of cytochrome C release. We have changed the colours and we hope this shows more clearly the lack of mitochondria in cells with extra centrosomes and treated with WEHI-539 and Q-VD-OPH . In addition, in Figure 4G, we have also included a picture of this cell with a “Fire LUT”. Here the high signal of mitochondria in a neighbouring cell can be easily perceived, while the cell without any mitochondria shows a complete lack of fluorescent signal. Since we have changed mitochondria to Cyan in Fig 4, we decided to change it in Fig 3, to maintain consistency in colours.

4. Line 200: "In both PLK4Ctl and PLK4OE treated with Carboplatin, the main phenotype was an increase in High mis-segregation divisions…(Fig 2A-B and F-G)". These panels don't show the incidence of mitotic phenotypes in the absence of carboplatin and so can't support this conclusion. The example "anaphase" in Fig S2D looks like it is in prometaphase. Though the cell shown has 1 acentric chromosome, it isn't clear how this will lead to high mis-segregation.

We are sorry that we did not make it clear that the controls- analysis of PLK4Ctl and PLK4OE can be found on Figs S1F and G for both the behaviours along the 72hrs, but also for the category of each mitotic defect. We have now made this clear in the text and in the Figure legends of Fig. 2A-B and F-G. Concerning the cell shown in Fig S2D, this is an anaphase. The spindle has the typical anaphase – elongated shape. Very different from prometaphase, where the longer axis is much shorter. We have spent many years characterizing the divisions of OVCAR8 cells, and this is certainly an anaphase. Please compare it with the S2H, where you can see this continuum of chromosomes between the two poles, typical of carboplatin treated cells.

5. The revised statement in the first paragraph of the discussion still misleadingly implies that HSET inhibitors have failed in clinical trials when they have not entered clinical trials. It also implies that Aurora A inhibitors have been tested in the clinic in an attempt to induce multipolar divisions when they were tested based on the ability to induce mitotic arrest. This section should be further revised.

We have changed this sentence, the reviewer is absolutely right in stating that the way we wrote this first paragraph was (even if without intention) misleading.

Minor comments

6. The chromosome bridge in Fig. 1A is not visible in the current version of the image.

In order to make the bridge more visible, we have changed the panels for "Fire Lut" and increased their size. We hope this makes things easier for the reader.

7. The statement on line 194-5, "Carboplatin treated cells have a reduction in proliferation compared to untreated cells (Fig. S1E)", does not appear to refer to Fig. S1E.
We think this is a mis-understanding, considering our intentions. Indeed the interphase length graph shown in S1E (top graph) shows that interphase length is extended in carboplatin treated cells. Further, the graphs shown in 2A-B, also referred to (in this sentence) show fewer cell cycles than in control cells. We have placed the reference S1E after Fig 2A-B.

8. An image of an "undefined" spindle in Fig S1I appears to be missing.
We are sorry about this confusing image. But the graph shows that there is no undefined spindle in control cells. This is why it is not shown. We have moved the word "undefined to the bottom panel.

9. At what time point were the data in Fig 3D-E collected? Fig 3A-B suggests this is likely to be the 48h timepoint, but I do not see this information in the text, figure or legend.
We are sorry that this was not mentioned in the Figure legend or text, but only in the methods. This data were collected at time point 72h. We have added this information in the Fig legend.

10. The IC50 values for WEHI-539 in PLK4OE and PLK4Ctrl cells appear to be switched in Fig 4A.
The IC50s are corrected, please note that in one case is uM and the other nM. PLK4OE cells are much more sensitive to WEHI-539 than non-induced cells.

11. How can 59% stage II patients be in the cohort comprising both High and Low CNR (Fig S7D, lines 569-570)?

Maybe our sentence is mis-leading? What this sentence means is that from our cohort- 59% of the patients have ovarian cancers in stage III. In other words, the majority are in stage III. And both high and low CNRs can be identified with the stage III patients. We have changed this sentence.

12. S7E and F have exactly the same p value (0.0182), which seems unlikely. Is this correct?

Yes, it is correct even if surprising.

13. The sentence on line 229-231 implies that No missegregation and slight mis-segregation events occur only in the first 24h of carboplatin treatment, and that other types of divisions do not occur during this time period. Is that correct?

The sentence the reviewer is refering to : "In particular around 35% of cell death was observed for the progeny of cells that had *No mis-segregation* or *Slight mis-segregation* defects, in contrast with PLK4Ctl where less than 10% of these cells died (Fig 2F, horizontal axis)".

We do not interpret this sentence as the reviewer. And as shown in Fig 2F, other types of cell division, including "high mis-segregation are also included.

We would be happy to change, but we were wondering if the reviewer is referring to another sentence?

14. Line 539 says STING knockout had no influence on sensitivity to WEHI-539 (Fig S6G-H), but Fig S6H indicates it causes a significant decrease in cell death.

In line 539, we do not mention Sting. The description of Sting is initiated in line 555. And here we write that: " We used a bulk LentiCRISPR knock-out approach of STING, but observed no influence on PLK4OE cells sensitivity to WEHI-539 (Fig S6G-H)". We were referring to the cells over-expressing PLK4OE. Is the reviewer referring to the data points of PLK4OE, WEHI-539 + sg SCRAMBLED and PLK4OE, WEHI-539 + sgSTING? Where the p-value is 0.0116? Even if statistically significant, we do not consider that this suggests a major role for the STING pathway. We would like to point out that on S6G, we show that sgSting is depleting the large majority of the protein and the differences in cell death remain very low.

15. On line 38, "occurs" really should be "can occur" because enhanced cell death is in some cases due to chromosome instability.

The reviewer is right. We have changed this sentence.

Reviewer #2: The authors have addressed the points raised in my initial review in Review Commons with the additional expts and addendums to the manuscript :

Reviewer 2, Major comments:

1. The authors state that (Line 133) "the increased multipolarity we observe in presence of the combined chemotherapy is caused by the effect of Paclitaxel on the capacity of cells to cluster centrosomes." Could the authors to back up this claim by reanalyzing the imaging data to look for
clustering as a survival mechanism versus inhibition of clustering in Paclitaxel-treated cells? Or
indeed test any of the range of available clustering inhibitors directly on PLK4OE and thus prove
the contribution of clustering to survival?

The experiments completed by the authors address in some way address my concerns about the contribution of centrosome clustering to cell survival in their model system and also show that Paclitaxel increases the incidence of multipolar/undefined mitotic spindles post-CA induction. In the final version, images representing "abnormally looking spindles that we

named undefined spindles" should also be included in this figure for clarity. Finally, reword "abnormally" to "abnormal".

We are sorry that this image is genertaing confusion. Reviewer1, had the same request. We actually show a "undefined" spindle in Fig S1I for PLK4OE + Paclitaxel. We define this figure as undefined, because there are three centrosomes that do not seem to participate in a clear mitotic spindle as microtubules are arranged in a way that does not allow to take conclusions in terms of spindle polarity. We did not show in control cells because these do not contain such spindles as shown in graph S1J. We have moved the word undefined to the respective image and we have also explained that we do not see this type of spindles in control cells in the Fig legend.

2. Fig 3: Results line 229-236 refer to quantification of fragmented nuclei which the authors interpret as poised for apoptosis. Micronuclei are also quantified- do the authors interpret this phenotype as advanced apoptosis? There is no mention of apoptotic bodies in the analysis. I would ask the authors to provide a bank of representative images with explanations to illustrate
their interpretation of the range of morphologies - differences between nuclear fragmentation, versus micronuclei versus DNA contained in apoptotic bodies.

The authors have adequately addressed my query by highlighting the presence of apoptotic bodies with additional labelling in Fig 3 and providing representative images of the various nuclear morphologies.

We thank this reviewer for his/her assessment of our revision.

3. Fig 6: While the authors have already acknowledged this as a weakness of the study, can the
patient data really be compared to cell line data on CA because inclusion of CNRs between 1.4
and 2 as "high CNR" is questionable given that this ratio represents a completely normal centrosome complement? Are the authors confident enough in the imaging technique that all centrosomes are being detected? Can the authors justify the inclusion of the 1.4-2 CNR tumours
by breaking down individual patient data on response to various treatments? Have the authors tried to analyse the cohort for OS and RFS using only those 9% of tumours exhibiting CA? What
does the analyses of Fig 6 and S6 look like with a CNR cut-off of 2 instead of 1.45? Does the reanalysis
show a better correlation between CNR and FIGO stage?

The authors have satisfactorily addressed all the queries raised in this comment.

We thank this reviewer for his/her assessment of our revision.

4. Although this patient cohort is described in a previous publication, authors should include a cohort description in a table within supplemental for this manuscript: age range of patients, number of patients in each stage, size of tumours, and most relevant to this study, treatment regimens- adjuvant versus neoadjuvant, surgery vs no surgery? How is the cohort selected-

sequentially
selected? inclusion/exclusion criteria?
Statement in abstract "we show that high centrosome numbers associate with improved chemo
responses" is too specific as we have no information on the treatment regimens received by the
patients (neo or adjuvant chemo versus surgical/radiological interventions?). Maybe treatment response would be more appropriate? Were there any cases of Pathological complete (or even near complete) response in this cohort and if so, what was the CNR in those cases?

The authors have satisfactorily addressed all the queries raised in this comment.

We thank this reviewer for his/her assessment of our revision.

5. The experimental PLK4 overexpression system is an accepted and clean method to induce CA
in vitro. Could the authors comment in the discussion on how they envision CA being induced as
a sensitizing agent in the clinical setting to support the translational aspects of their work?

The authors have satisfactorily answered the query raised in this comment.
We thank this reviewer for his/her assessment of our revision.

Reviewer 2, Minor comments:
Line 54: Suggest rephrasing of the statement “and this can be favored by centrosome amplification (29)”
Perhaps a word like potentiated instead of favored?
Line 67: Again consider using an alternative to favored “We show that centrosome amplification favors the response to combined Carboplatin and Paclitaxel via multiple mechanisms.”
Favored is in fact used throughout the manuscript text- in my opinion this is not a scientific enough term and would consider replacing with alternative.
Line 263: “Centrosome amplification primes for MOMP and sensitizes cells to a diversity of chemotherapies.” CA primes to one very specific BCL-XL inhibitor in this section so consider modifying the title of the section.

The authors have satisfactorily addressed all the queries raised in the minor comments section.

We thank this reviewer for his/her assessment of our revision.
