# Supplementary material for: Centrosome amplification primes ovarian cancer cells for apoptosis and potentiates the response to chemotherapy
Source: PLoS Biol. 2024 Sep 5;22(9):e3002759. doi: 10.1371/journal.pbio.3002759 (PMC11441705; doi:10.1371/journal.pbio.3002759)
Supplement: Attachment — Submitted filename: Response to reviewers_Edwards et al 2023.docx [file pbio.3002759.s032.docx]

**Manuscript number:** RC-2023-02119

**Corresponding author(s):** Renata Basto

*[The “revision plan” should delineate the revisions that authors intend to carry out in response to the points raised by the referees. It also provides the authors with the opportunity to explain their view of the paper and of the referee reports.*

*The document is important for the editors of affiliate journals when they make a first decision on the transferred manuscript. It will also be useful to readers of the reprint and help them to obtain a balanced view of the paper.*

*If you wish to submit a full revision, please use our "*[*Full Revision*](https://reviewcommons.org/templates/full-revision.docx)*" template.* ***It is important to use the appropriate template to clearly inform the editors of your intentions.****]*

***Update January 2024: Response to reviewers about the performed experiments are in orange.***

## 1. General Statements [optional]

We thank the reviewers for their comments, and their appreciation of the value and thoroughness of our work in identifying a novel and clinically relevant consequence of centrosome amplification in favoring cell death. The reviewers accurately identify weaknesses of our work which we had also pointed out in the discussion of our manuscript. In particular, we agree as pointed out by Reviewer 1 that direct links between our cellular and clinical observations are difficult to establish given the low level of centrosome amplification observed in the tumor samples. Although multiple hypothesis might explain why preferentially eliminating a small population of cells is beneficial for the patients, we consider that this is out of the scope of this manuscript. However, given that our cellular and clinical observations point in the same direction, we remain confident in the value of presenting them together in this manuscript. We have made it clearer both in the results section and discussion that further work is required to better understand the influence of low levels of centrosome amplification on chemotherapy responses in patients.

We also thank the reviewers for their suggestions to improve the *in vitro* work we have performed. Our point by point response below lists the experiments we plan to perform, and corrections we have already included in this submitted version. Although Reviewer 3 points out that the molecular mechanism underlying apoptotic priming in cells with centrosome amplification remains a mystery, we argue that the identification of this priming already provides a mechanistic explanation for enhanced chemotherapy responses. Our careful and thorough analysis of these responses, using a diversity of advanced technical approaches was key to achieve this. We were also able to clearly rule out that priming is caused by a previously characterized centrosome amplification consequence, demonstrating its novelty. Reviewer 3’s characterization of our work as “archival” is diminishing to say the least, and we believe multiple aspects of this work will be built upon, even beyond the identification of a molecular mechanism which will of course also be important. Indeed, centrosome amplification is observed in a diversity of healthy cell types (megakaryocytes, B cells, hepatocytes…) and could contribute to the homeostasis of these tissues via apoptotic priming. We predict the translational perspectives of this work also to be important, from the point of view of centrosome amplification in disease, but also in understanding apoptotic priming and responses to BH3-mimetics.

## 2. Description of the planned revisions

Reviewer 1, Major comments:

3. The conclusion that centrosome amplification primes to apoptosis irrespective of mitotic defects is largely based on low resolution timelapse analysis (20x magnification, 10 minute imaging intervals, no tubulin). Imaging at this resolution is likely to miss mitotic defects, reducing the confidence with which this conclusion can be drawn.

We were unsure of the exact point brought up by Reviewer 1 here and we have consulted Reviewer 1 (through Reviews commons) to confirm the revision plan. In Figure 5A, we show that the level of heterogeneity stemming from chromosome instability is lower for PLK4OE than for MPS1 inhibition, and Figure 5D then shows that apoptotic priming only occurs in PLK4OE, and not in response to MPS1 inhibition. Combined, these experiments allow us to conclude that apoptotic priming occurs independently of mitotic defects. Nevertheless, we propose to reproduce the live-imaging of mitosis, increasing the resolution and including tubulin, to better visualize mitotic defects in response to the different mitotic perturbations induced.

We have performed the imaging at higher spatio-temporal resolution (40x, 2,5min intervals, 1μM z-sampling), and included the information in Fig S2F-G. We were however not able to satisfyingly perform live imaging of spindles, as addition of increasing concentrations of SPY-tubulin (a taxane derivative) required to visualize spindles decreased bipolar divisions in PLK4OE cells (See figure below). The data presented therefore does not include information about spindles via live imaging.

Focusing on chromosome behaviors, the imaging performed at higher resolution did not reveal any yet unobserved behavior in PLK4OE cells (data presented in Fig S2F-G). This data is therefore not conflicting with our previous conclusion that cell death occurs at higher levels in PLK4OE than in PLK4Ctl following similar mitotic phenotypes. We were therefore comforted in these conclusions and strengthened this point in our manuscript.

4. Data from timelapse analysis of DNA content in Fig. 2 are used to conclude that Plk4OE cells are more sensitive to carboplatin due to mitotic defects that occurred without multipolar spindles. However, it is premature to conclude that multipolar spindles were not involved in DNA mis-segregation without visualizing the spindles themselves. While DNA positioning can be used as a proxy for spindle morphology, as performed here, it only reliably detects multipolar spindles when all poles are relatively equal in size and the multipolar spindle is maintained throughout mitosis. However, the poles in multipolar spindles often differ in size and ability to recruit DNA. Additionally, they often cluster over time, which can preclude their identification when only visualizing DNA, especially at 20x magnification. Compelling evidence that high mis-segregation is occurring without multipolar spindles would require visualizing the spindles and also demonstrating the cause of the increased chromosome mis-segregation. (Are acentric fragments being mis-segregated as lagging chromosomes?)

We agree with Reviewer 1 that the “High mis-segregation” mitotic phenotype we describe is poorly characterized in our original manuscript, and that we cannot formally exclude that multipolar spindles are involved, although we observe this phenotype in similar proportions in PLK4Ctl and PLK4OE. We also agree that identifying the origin of increased chromosome mis-segregation is relevant here. We therefore propose to characterize spindles and mis-segregating chromosomes by imaging fixed mitotic figures upon Carboplatin treatment, staining for Tubulin, centrosomes, and centromeres. This will allow us to better determine if Carboplatin induces the same mitotic phenotypes in PLK4OE and PLK4Ctl cells.

To answer this point, we performed imaging of spindles in cells fixed after 48h of Carboplatin exposure, and the data is presented in Fig S2A-E. We are able to confirm that the High mis-segregation phenotypes we observe occur on spindles that are mainly bipolar based on the tubulin staining. However as shown in the figures, despite spindles being bipolar, additional microtubule organizing centers (MTOCs) are observed even in PLK4Ctl cells. Importantly not all these MTOCs contribute to generating spindle poles. They also differ in their organization from PCM and centrioles. Interestingly, in *Drosophila* neuroblasts, centrosome amplification also leads to this type of bipolar spindle with additional MTOCs localised within the spindle. We report this information but do not aim to explain it further as it is out of the scope of this study. We therefore mainly draw the conclusion that the High mis-segregation phenotype is associated with bipolar spindles. Additionally, as suggested by the reviewer, we stained for centromeres and indeed observed frequent occurrence of acentric chromosomes (Fig. S2D-E). Together these observations strengthen the idea that this type of mitosis may result in lethal levels of aneuploidy in both PLK4Ctl and PLK4OE OVCAR8 cells.

5. The images in Fig. 3D and 4D are not of sufficient resolution to support the central conclusion that centrosome amplification primes cells for MOMP. This conclusion is further weakened by the facts that 1) Plk4OE was the only source of centrosome amplification tested and 2) Plk4OE was reported to prime for MOMP in only 2 of 3 cell lines. Potential explanations for the lack of priming in SKOV3 cells should be discussed. Additionally, the sensitization in Fig. S4H-J appears quite modest. (These data are also difficult to see, perhaps because the Plk4Ctl -/+ chemo conditions are overlapping.)

We have made images in Fig. 3D and 4D larger. We hope that this makes our observations of Cytochrome C release (quantified in Fig. 3E and 4E) easier to visualize for readers. We would like to point out that our conclusion that centrosome amplification primes for MOMP in OVCAR8 does not only arise from the assays presented in these figures. In Figures 4A and 4C we show by MTT assays and detection of apoptotic cells by cytometry respectively, that PLK4OE OVCAR8 are sensitized to BH3-mimetic WEHI-539 compared to PLK4Ctl cells. We also show this priming using BH3-mimetic Navitoclax in Fig S4F. Regarding the source of centrosome amplification, we use OVCAR8 cells devoid of the inducible PLK4OE transgene and show that “natural” centrosome amplification also makes OVCAR8 cells more sensitive to WEHI-539 (Fig. S4C). This strongly suggests that priming does indeed stem from centrosome amplification and not from other consequences of PLK4 over-expression. Nevertheless we are currently generating cells to induce centrosome amplification via SAS6 over-expression, to test an alternative source of centrosome amplification.

Unfortunately, the system we generated to over-express SAS6 (SAS6 Delta KEN box) was not successful in inducing centrosome amplification (presented in Fig S5C). At this stage we have not determined whether this is because of lack of protein accumulation, or differential consequences of SAS6 accumulation on centriole biogenesis in OVCAR8 from what was previously described. However we remain confident in the importance of the apoptotic priming we describe as we also observe it for naturally occurring centrosome amplification in the parental OVCAR8 cell line.

We do not claim that this apoptotic priming is a universal consequence of centrosome amplification, as indeed we show that it is not observed for the cell line SKOV3 (Fig. S4F). For now, we do not have a clear hypothesis of the reason for the cell line differences. Initially we hypothesized that p53 status could be in cause because OVCAR8 and COV504 both express mutant p53 whereas p53 protein is completely absent in SKOV3. However we observe that p53 does not seem to affect cell death signaling in OVCAR8 (Fig. S3C-D), making this hypothesis less likely. The 3 cell lines are indeed very different in terms of origin and genomic alterations, making it difficult at this stage to propose an evidence-based explanation of differences in terms of apoptotic priming.

Finally, regarding the sensitization to chemotherapy associated priming presented in Fig. S4H-J, we have made the figures bigger and non-overlapping hoping that this makes them easier to read. Additionally, we agree that the effect of centrosome amplification appears modest. The trypan-blue assays we used have the advantage of being relatively high-throughput, but they only detect a fraction of the killed cells: only cells that are late in the apoptotic process but that haven’t yet been degraded. This makes the assay less sensitive. The general tendency we observe nevertheless proposes an association between apoptotic priming induced by centrosome amplification, and an enhanced sensitivity to a diversity of chemotherapy agents. We propose to confirm this for OVCAR8 cells treated with Olaparib, by performing cytometry experiments staining for AnnexinV and Propidium Iodide in order to increase sensitivity by detecting earlier signs of apoptosis.

We performed flow cytometry to quantify the proportion of Annexin V positive cells in response to Olaparib, and indeed observe that PLK4OE cells present enhanced apoptosis in response to this drug compared to PLK4Ctl cells. This data is presented in Fig 4H.

Reviewer 2, Major comments:

1. The authors state that (Line 133) "the increased multipolarity we observe in presence of the combined chemotherapy is caused by the effect of Paclitaxel on the capacity of cells to cluster centrosomes." Could the authors to back up this claim by reanalyzing the imaging data to look for clustering as a survival mechanism versus inhibition of clustering in Paclitaxel-treated cells? Or indeed test any of the range of available clustering inhibitors directly on PLK4OE and thus prove the contribution of clustering to survival?

We agree that the conclusion that Paclitaxel suppresses centrosome clustering is not sufficiently backed by experimental data. We cannot directly view clustering in the live-imaging experiments we performed, because we are visualizing neither tubulin nor centrosomes. To clarify this point, we will:

1. Image fixed mitotic figures of Plk4Ctl and PLK4OE cells untreated or treated with Paclitaxel, staining for tubulin and centrosomes to identify if indeed Paclitaxel increases the proportion of anaphases with multiple poles characterized by the presence of centrosomes. We will use this type of assay as live imaging approaches to film both microtubules and centrosomes will not be feasible within the timing of this revision, but also because paclitaxel responses maybe modified if tubulin dyes are used such as Sir-tubulin.

2. We will use HSET inhibitor CW069, to test if this also prevents centrosome clustering.

3. If this is the case, we will then test if CW069 also preferentially kills PLK4OE compared to PLK4Ctl by Trypan-blue viability assays.

1. We fixed PLK4OE cells upon 24h of Paclitaxel treatment and observed spindles using an antibody against α-Tubulin, PCM with an antibody against CEP192, and centrioles with an antibody against Centrin 3. Anaphases were very infrequent in this experimental set-up, most likely owing to this mitotic phase’s fast completion. We therefore focused on prometaphase and metaphase cells. In untreated PLK4OE cells we observed very few cells with complete centrosome clustering into two groups. The dominating phenotype was pseudo-bipolar spindles where the Tubulin and DNA was organized in a bipolar fashion, but with additional MTOCs organized around unclustered centrosomes. This phenotype was largely absent when cells were treated with Paclitaxel where multipolar spindles were the most frequent. In view of the incomplete clustering observed in this experiment, we removed the reference to clustering in our results section and focus on Paclitaxel as a source of spindle multipolarity. This data is presented in Fig S1 I-J.

2. We first determined the IC50 of CW069 in OVCAR8 using MTT viability assays and found values around 100μM. We therefore subsequently used this concentration in live-imaging experiments to test if this compound induces multipolar divisions. We observed no increase in multipolarity in PLK4OE cells treated with CW069. However we observe a strong extension of time cells spend in mitosis. This suggests that in OVCAR8, this HSET inhibitor induces cell death by extending time in mitosis rather than by inducing high levels of aneuploidy on multipolar spindles. Although this observation is interesting, we propose not to include it in this manuscript as it is not related to the apoptotic priming which is our main focus here. This data is presented in the figures below.

3. Description of the revisions that have already been incorporated in the transferred manuscript

Reviewer 1, Major comments:

1. In its current form, the title suggests that the major role of centrosome amplification in sensitizing to chemotherapy is independent of multipolar divisions. Based on Figure 1, this is misleading. Figure 1D shows that in centrosome amplified cells treated with combination chemotherapy, the most common cause of death is high mis-segregation on multipolar spindles. Modifying the title to "Centrosome amplification favors the response to chemotherapy in ovarian cancer by priming for apoptosis in addition to promoting multipolar division" would more accurately reflect the data.

We agree with Reviewer 1 that our title should include the promotion of multipolar divisions, and have modified the title accordingly.

6. Line 191 points out that more Plk4OE cells that were in G1 at the beginning of carboplatin died than Plk4Ctl cells. However, in Fig. 2H-I, it looks like longer G1 durations in the presence of carboplatin led to increased cell death and that the Plk4OE cells happened to spend more time in G1 at the beginning of carboplatin treatment than Plk4Ctl cells did. Is this the case? Quantification of the average time spent in G1 for each group would be helpful.

Upon Carboplatin exposure, G1 length is indeed longer for PLK4OE cells compared to PLK4Ctl cells, as shown in Fig. S2D for complete G1 phases observed after the first mitosis (although the induced lengthening is mild compared to the observed extension of G2 induced upon carboplatin exposure shown in Fig. S2D). The same tendency, although not significant, is observable for cells in G1 at the beginning of carboplatin treatment (see figure below), although it is harder to conclude because these are not complete G1 phases.

To assess links between G1 phase length and cell fate, we have plotted below the length of G1 depending on whether cells live or die. For cells in G1 at the onset of Carboplatin treatment, longer times in G1 are observed for cells that die. However, this link between G1 length and cell fate is not observed in the 2^nd^ generation. We do not have an explanation for this discrepancy, but we are more inclined to rely on the analysis of cells in the 2^nd^ generation for which we can access complete G1 lengths. Given that increased cell death is observed for PLK4OE mainly in the 2^nd^ generation (Fig. 2E), we therefore do not have sufficient grounds to propose a link between centrosome amplification, G1 lengthening, and subsequent consequences on cell fate. We have added the analysis of G1 length depending on cell fate in the second generation as Fig. S1E.

Reviewer 1, minor comments:

7. The authors cite Fig. 1B when drawing the conclusion that "combined chemotherapy induced a stronger reduction of viable cells produced per lineage in PLK4OE compared to PLK4Ctl". But Fig. 1B shows that combination chemotherapy produced a similar decrease in viable cells per lineage +/- Plk4OE. If anything, the Plk4OE+ cells showed slightly less sensitivity because they proliferated more poorly in the absence of chemotherapy. This is also true for carboplatin sensitivity in Fig. 2D (line 156).

Here our focus is actually more on the proportion of cell death that on the number of viable cells. We agree that the way we wrote this makes it confusing so we have re-written this paragraph to make it clearer.

8. Line 202 concludes that Fig. S2H-I shows that Plk4OE doesn't affect recruitment of DNA damage repair factors. The dotted outlines around the nuclei in Fig. S2H-1 make it very difficult to see, but it appears that gH2AX, FancD2, and 53BP1 signals are lower in Plk4OE cells.

We have made images in Fig. S2H bigger and the dotted outlines around nuclear less strong, and we hope this makes the signal easier to see. Strong cell to cell variations in signal make it hard to draw conclusions from images, although we have aimed to present this heterogeneity. The quantifications shown in Fig. S2I however show that there are no differences in Rad51 or FANCD2 recruitment in PLK4OE cells. For 53BP1 however, we observed less recruitment in PLK4OE for one biological replicate (squares in quantification shown in Fig. S2I) but not in the two other replicates. Although there may be some interesting observation here, this difference does not appear sufficiently robust to consider it as relevant in the context of this study.

9. The images for "dies in interphase" and "dies in mitosis" in Fig. 1B are suboptimal. Alternative images would be beneficial.

We have modified the images and added timepoints to make the phenotypes clearer. We have also added supplementary movies to better visualize the events (See Movie S1A for cell death events).

10. It would be helpful to discuss the clinical relevance of WEHI-539 and Navitoclax.

We have further developed the section of our discussion about the clinical relevance of BH3-mimetics and these drugs.

11. The discussion states that "mitotic drugs that limit centrosome clustering have had limited success in the clinic". I am not aware of any drugs that limit centrosome clustering that are suitable for in vivo use and the citation provided does not mention centrosome clustering.

We thank Reviewer 1 for this comment. Indeed, we oversimplified things a bit here, and have rewritten this paragraph. We have however kept the citation because although this reference does not directly mention centrosome clustering, some of the discussed drugs have been shown to kill cancer cells via centrosome unclustering *in vitro* in other studies.

12. The dark purple and black are very difficult to discriminate (Figure 1,2 and S1), as are the light green and light turquoise (Fig. 4A,S4A-B, S4F, S4H).

We changed these colors in the indicated graphs, and also in other figures where they were used. We hope these changes make the figures easier to read.

13. Line 246 claims that Fig. S3B shows that p21 and PUMA mildly increase upon carboplatin exposure, but it isn't clear that these increase in a biologically or statistically significant manner.

We have modified this paragraph because indeed it seems unlikely that the differences are statistically or biologically significant.

14. The green used to indicate S/G2 in Fig. S2A-B is different in Plk4 Ctrl vs Plk4OE cells.

We thank Reviewer 1 for spotting this and have changed the colors.

15. I do not believe that carboplatin + paclitaxel is standard of care treatment for breast or lung cancer, as stated on line 48-49.

Based on the guidelines of the NIH, we believe that these two drugs are indeed used in combination for the treatment of Stage IV non small cell lung cancer, as well as triple-negative breast cancer. (<https://www.cancer.gov/types/lung/hp/non-small-cell-lung-treatment-pdq#_48414_toc>, <https://www.cancer.gov/types/breast/hp/breast-treatment-pdq#_1049>).

However, given the complexity and diversity of treatment protocols, we have modified the text so as not to convey the idea that these are the only drugs used.

16. This study advances, but does not complete our understanding of centrosome amplification in breast cancer, as stated on line 75.

Agreed and changed.

17. Line 297 describes Navitoclax as an "inhibitor of BCL2, BCL-XL and BCL2". (ie BCL2 is listed twice).

Thank you for noticing this, we have corrected this.

18. It's not clear why line 120, which refers to effects of combined chemotherapy, cites Fig. S1G-I, which apparently show data from untreated (even without dox?) Plk4Ctrl and Plk4OE cells.

We indeed meant to refer to Fig. 1E-F and have therefore made this change.

19. In Fig. S6A, how can the mitotic index be 200%?

We thank Reviewer 1 for noticing the poor labelling of this figure. It is not a percentage of cells we are presenting, but the number of mitotic figures identified in 10 analyzed fields. We have corrected the figure.

Reviewer 2 major comments:

2. Fig 3: Results line 229-236 refer to quantification of fragmented nuclei which the authors interpret as poised for apoptosis. Micronuclei are also quantified- do the authors interpret this phenotype as advanced apoptosis? There is no mention of apoptotic bodies in the analysis. I would ask the authors to provide a bank of representative images with explanations to illustrate their interpretation of the range of morphologies - differences between nuclear fragmentation, versus micronuclei versus DNA contained in apoptotic bodies.

The cells we defined as “poised for apoptosis” are cells that release cytochrome C in presence of a pan-caspase inhibitor. These cells are therefore activating mitochondrial outer membrane permeabilization without executing apoptosis. It is then within these cells that we observe different nuclear morphologies, reflecting different behaviors in mitosis rather than apoptosis advancement. Apoptotic bodies are not observed in these cells, because they are actually not executing apoptosis owing to the presence of the pan-caspase inhibitor. We visualized apoptotic bodies only in absence of pan-caspase inhibitor. These are indicated by white arrow-heads, in Fig. 3D which was made bigger for more clarity. We have indicated on panels in Fig. 3D the different nuclear morphologies quantified in Fig. 3F.

4. Although this patient cohort is described in a previous publication, authors should include a cohort description in a table within supplemental for this manuscript: age range of patients, number of patients in each stage, size of tumours, and most relevant to this study, treatment regimens- adjuvant versus neoadjuvant, surgery vs no surgery? How is the cohort selected- sequentially selected? inclusion/exclusion criteria?
Statement in abstract "we show that high centrosome numbers associate with improved chemo responses" is too specific as we have no information on the treatment regimens received by the patients (neo or adjuvant chemo versus surgical/radiological interventions?). Maybe treatment response would be more appropriate? Were there any cases of Pathological complete (or even near complete) response in this cohort and if so, what was the CNR in those cases?

We have included the cohort description in Supplementary Table 1. This is a retrospective cohort, so no specific inclusion criteria were applied. Treatment mainly consisted of surgery (100% patients) followed by adjuvant chemotherapy consisting of platinium salts and/or taxanes (84% of patients, 67% treated with both). Despite the wide common ground of treatment (surgery followed by taxanes and/or platinium salts for 84% of patients), we have nevertheless modified the abstract as suggested by Reviewer 2. Regarding complete or near-complete response, there were no such cases in this cohort.

Reviewer 2, minor comments:

Just some minor points on language:
Line 54: Suggest rephrasing of the statement "and this can be favored by centrosome amplification (29) "
Perhaps a word like potentiated instead of favored?
Line 67: Again consider using an alternative to favored "We show that centrosome amplification favors the response to combined Carboplatin and Paclitaxel via multiple mechanisms."
Favored is in fact used throughout the manuscript text- in my opinion this is not a scientific enough term and would consider replacing with alternative.

We have replaced the term favor with more appropriate terms, depending on the context.

## 4. Description of analyses that authors prefer not to carry out

Reviewer 1, major comments:

2. In a previous technical tour de force (Morretton et al, EMBO Mol Med 2022), the Basto lab quantified centriole numbers in the ovarian patient cancer samples analyzed here, and found that the percentage of cells with centrosome amplification in a given ovarian tumor is quite small, only reaching a maximum of 3.2%. It is critical background information to cite that quantification here. This information also begs the question of whether introducing this low rate of centrosome amplification is sufficient to cause a more global apoptotic priming in the sample, as suggested.

We have now included this important background information in our results section. We agree with Reviewer 1 that the low levels of centrosome amplification in tumors may not cause a more global apoptotic priming in the whole tumor. However, based on our findings, this low proportion of cells will most likely be more sensitive to chemotherapy. We cannot affirm for now what will be the consequences of the preferential elimination of these cells. However, given centrosome amplification’s potential to promote malignant behaviors such as genetic instability or invasiveness, we hypothesize that the elimination of these cells may have effects on tumor survival that are not proportional to their numbers. Testing this hypothesis would require many more experiments using *in vivo* models, which we cannot carry out within the scope of this study.

Reviewer 2, major comments:

3. Fig 6: While the authors have already acknowledged this as a weakness of the study, can the patient data really be compared to cell line data on CA because inclusion of CNRs between 1.4 and 2 as "high CNR" is questionable given that this ratio represents a completely normal centrosome complement? Are the authors confident enough in the imaging technique that all centrosomes are being detected? Can the authors justify the inclusion of the 1.4-2 CNR tumours by breaking down individual patient data on response to various treatments? Have the authors tried to analyse the cohort for OS and RFS using only those 9% of tumours exhibiting CA? What does the analyses of Fig 6 and S6 look like with a CNR cut-off of 2 instead of 1.45? Does the re-analysis show a better correlation between CNR and FIGO stage?

At the single-cell level, centrosome amplification is indeed defined as the presence of more than 2 centrosomes per cell. Tumor samples are characterized by heterogeneous centrosome numbers, with some regions showing extensive centrosome loss, and some others showing nuclei associated with either one, two or various levels of centrosome amplification. In such a heterogeneous population of cells, it is therefore not straight-forward to use the cut-off CNR=2 to define tumors with centrosome amplification. Here, we nevertheless present for the reviewer the analysis of clinical parameters using the cut-off for CNR at 2 as proposed. Using this cut-off, High CNR patients still show improved overall survival, but a non-statistically significant extension of time to relapse. There are very few patients with CNR>2, and therefore we remain unconvinced by the statistical value of such an analysis.

The definition of a cut-off at 1.45 was not arbitrary. We dichotomized the population into two groups using the Classification And Regression Trees (CART) method. Taking into consideration the binary outcome “relapse within 6 months or no relapse within 6 months”  this method resulted in the categorization of the cohort into low CNR (≤ 1.45) and high CNR (> 1.45). Independently, we also used predictiveness curves to estimate an optimal cut-off parameter for a continuous biomarker such as the CNR. The threshold obtained by this robust methodology was in agreement with CART approach with a cut-off of 1.40 (see figure below).

Dichotomizing the population does not guarantee the identification of significant clinical differences between the generated groups. We therefore analyzed overall survival and time to relapse *ex post,* and observed that high CNR and low CNR populations differed significantly for both these parameters.

Regarding FIGO stage, given frequent late detection of ovarian cancer, 59% of the cohort is considered at stage III (See Fig. S6C, reproduced below). All patients with CNR>2 are in the group of Stage III, except one which is Stage II. However, no patient with CNR>2 is in Stage IV, arguing that even with a higher CNR cut-off, there is no association between CNR and Figo stage.

5. The experimental PLK4 overexpression system is an accepted and clean method to induce CA in vitro. Could the authors comment in the discussion on how they envision CA being induced as a sensitizing agent in the clinical setting to support the translational aspects of their work?

In the clinical setting, we do not suggest to induce centrosome amplification as a sensitization agent. Indeed, centrosome amplification induces multiple phenotypes associated with malignancy (genomic instability, invasiveness). The translational aspects of our work relate more to the detection of centrosome amplification as a potential biomarker of chemotherapy responses, from conventional chemotherapies to BH3-mimetics for which biomarkers are absent. This aspect we have commented on in our discussion.

Reviewer 2, minor comments:

Line 263: "Centrosome amplification primes for MOMP and sensitizes cells to a diversity of chemotherapies." CA primes to one very specific BCL-XL inhibitor in this section so consider modifying the title of the section.

We agree that centrosome amplification makes cells sensitive to a specific BCL-XL inhibitor. However, we nevertheless claim that this very specific priming, can potentiate these cells responses to a diverse range of chemotherapies with different targets (paclitaxel, carboplatin, and olaparib).
