# Supplementary material for: Centrosome amplification primes ovarian cancer cells for apoptosis and potentiates the response to chemotherapy
Source: PLoS Biol. 2024 Sep 5;22(9):e3002759. doi: 10.1371/journal.pbio.3002759 (PMC11441705; doi:10.1371/journal.pbio.3002759)
Supplement: Attachment — Submitted filename: PBIOLOGY-D-23-03107R2.Reviewer2.docx [file pbio.3002759.s033.docx]

***Plos Biology Review SHORT REPORT: Revision Plan from Review Commons***

***Manuscript number: RC-2023-02119***

***Corresponding author(s): Renata Basto***

**The authors have addressed the points raised in my initial review in Review Commons with the additional expts and addendums to the manuscript :**

Reviewer 2, Major comments:

1. *The authors state that (Line 133) "the increased multipolarity we observe in presence of the*

*combined chemotherapy is caused by the effect of Paclitaxel on the capacity of cells to cluster*

*centrosomes." Could the authors to back up this claim by reanalyzing the imaging data to look for*

*clustering as a survival mechanism versus inhibition of clustering in Paclitaxel-treated cells? Or*

*indeed test any of the range of available clustering inhibitors directly on PLK4OE and thus prove*

*the contribution of clustering to survival?*

The experiments completed by the authors address in some way address my concerns about the contribution of centrosome clustering to cell survival in their model system and also show that Paclitaxel increases the incidence of multipolar/undefined mitotic spindles post-CA induction. In the final version, images representing “abnormally looking spindles that we named undefined spindles” should also be included in this figure for clarity. Finally, reword “abnormally” to “abnormal”

2. *Fig 3: Results line 229-236 refer to quantification of fragmented nuclei which the authors*

*interpret as poised for apoptosis. Micronuclei are also quantified- do the authors interpret this*

*phenotype as advanced apoptosis? There is no mention of apoptotic bodies in the analysis. I*

*would ask the authors to provide a bank of representative images with explanations to illustrate*

*their interpretation of the range of morphologies - differences between nuclear fragmentation,*

*versus micronuclei versus DNA contained in apoptotic bodies.*

The authors have adequately addressed my query by highlighting the presence of apoptotic bodies with additional labelling in Fig 3 and providing representative images of the various nuclear morphologies.

3. *Fig 6: While the authors have already acknowledged this as a weakness of the study, can the*

*patient data really be compared to cell line data on CA because inclusion of CNRs between 1.4*

*and 2 as "high CNR" is questionable given that this ratio represents a completely normal*

*centrosome complement? Are the authors confident enough in the imaging technique that all*

*centrosomes are being detected? Can the authors justify the inclusion of the 1.4-2 CNR tumours*

*by breaking down individual patient data on response to various treatments? Have the authors*

*tried to analyse the cohort for OS and RFS using only those 9% of tumours exhibiting CA? What*

*does the analyses of Fig 6 and S6 look like with a CNR cut-off of 2 instead of 1.45? Does the reanalysis*

*show a better correlation between CNR and FIGO stage?*

The authors have satisfactorily addressed all the queries raised in this comment.

*4. Although this patient cohort is described in a previous publication, authors should include a*

*cohort description in a table within supplemental for this manuscript: age range of patients,*

*number of patients in each stage, size of tumours, and most relevant to this study, treatment*

*regimens- adjuvant versus neoadjuvant, surgery vs no surgery? How is the cohort selected- sequentially*

*selected? inclusion/exclusion criteria?*

*Statement in abstract "we show that high centrosome numbers associate with improved chemo*

*responses" is too specific as we have no information on the treatment regimens received by the*

*patients (neo or adjuvant chemo versus surgical/radiological interventions?). Maybe treatment*

*response would be more appropriate? Were there any cases of Pathological complete (or even*

*near complete) response in this cohort and if so, what was the CNR in those cases?*

The authors have satisfactorily addressed all the queries raised in this comment.

5. *The experimental PLK4 overexpression system is an accepted and clean method to induce CA*

*in vitro. Could the authors comment in the discussion on how they envision CA being induced as*

*a sensitizing agent in the clinical setting to support the translational aspects of their work?*

The authors have satisfactorily answered the query raised in this comment.

*Reviewer 2, Minor comments:*

*Line 54: Suggest rephrasing of the statement “and this can be favored by centrosome amplification (29)”*

*Perhaps a word like potentiated instead of favored?*

*Line 67: Again consider using an alternative to favored “We show that centrosome amplification favors the response to combined Carboplatin and Paclitaxel via multiple mechanisms.”*

*Favored is in fact used throughout the manuscript text- in my opinion this is not a scientific enough term and would consider replacing with alternative.*

*Line 263: “Centrosome amplification primes for MOMP and sensitizes cells to a diversity of chemotherapies.” CA primes to one very specific BCL-XL inhibitor in this section so consider modifying the title of the section.*

The authors have satisfactorily addressed all the queries raised in the minor comments section.
